# Comparison of Conventional Lipoprotein Tests and Apolipoproteins in the Prediction of Cardiovascular Disease: Data From UK Biobank

**DOI:** 10.1161/CIRCULATIONAHA.119.041149

**Published:** 2019-06-20

**Authors:** Claire Welsh, Carlos A. Celis-Morales, Rosemary Brown, Daniel F. Mackay, James Lewsey, Patrick B. Mark, Stuart R. Gray, Lyn D. Ferguson, Jana J. Anderson, Donald M. Lyall, John G. Cleland, Pardeep S. Jhund, Jason M.R. Gill, Jill P. Pell, Naveed Sattar, Paul Welsh

**Affiliations:** 1 Institute of Cardiovascular and Medical Sciences (C.W., C.A.C.-M., R.B., P.B.M., S.R.G., L.D.F., P.S.J., J.M.R.G., N.S., P.W.), University of Glasgow, United Kingdom.; 2 Institute of Health and Wellbeing (D.F.M., J.L., J.J.A., D.M.L., J.G.C., J.P.P.), University of Glasgow, United Kingdom.

**Keywords:** apolipoprotein A-1, apolipoprotein B, cardiovascular diseases, cholesterol, HDL, cholesterol, LDL

## Abstract

**Background:**

Total cholesterol and high-density lipoprotein cholesterol (HDL-C) measurements are central to cardiovascular disease (CVD) risk assessment, but there is continuing debate around the utility of other lipids for risk prediction.

**Methods:**

Participants from UK Biobank without baseline CVD and not taking statins, with relevant lipid measurements (n=346 686), were included in the primary analysis. An incident fatal or nonfatal CVD event occurred in 6216 participants (1656 fatal) over a median of 8.9 years. Associations of nonfasting lipid measurements (total cholesterol, HDL-C, non–HDL-C, direct and calculated low-density lipoprotein cholesterol [LDL-C], and apolipoproteins [Apo] A1 and B) with CVD were compared using Cox models adjusting for classical risk factors, and predictive utility was determined by the C-index and net reclassification index. Prediction was also tested in 68 649 participants taking a statin with or without baseline CVD (3515 CVD events).

**Results:**

ApoB, LDL-C, and non–HDL-C were highly correlated (*r*>0.90), while HDL-C was strongly correlated with ApoA1 (*r*=0.92). After adjustment for classical risk factors, 1 SD increase in ApoB, direct LDL-C, and non–HDL-C had similar associations with composite fatal/nonfatal CVD events (hazard ratio, 1.23, 1.20, 1.21, respectively). Associations for 1 SD increase in HDL-C and ApoA1 were also similar (hazard ratios, 0.81 [both]). Adding either total cholesterol and HDL-C, or ApoB and ApoA, to a CVD risk prediction model (C-index, 0.7378) yielded similar improvement in discrimination (C-index change, 0.0084; 95% CI, 0.0065, 0.0104, and 0.0089; 95% CI, 0.0069, 0.0109, respectively). Once total and HDL-C were in the model, no further substantive improvement was achieved with the addition of ApoB (C-index change, 0.0004; 95% CI, 0.0000, 0.0008) or any measure of LDL-C. Results for predictive utility were similar for a fatal CVD outcome, and in a discordance analysis. In participants taking a statin, classical risk factors (C-index, 0.7118) were improved by non–HDL-C (C-index change, 0.0030; 95% CI, 0.0012, 0.0048) or ApoB (C-index change, 0.0030; 95% CI, 0.0011, 0.0048). However, adding ApoB or LDL-C to a model already containing non–HDL-C did not further improve discrimination.

**Conclusions:**

Measurement of total cholesterol and HDL-C in the nonfasted state is sufficient to capture the lipid-associated risk in CVD prediction, with no meaningful improvement from addition of apolipoproteins, direct or calculated LDL-C.

## Abstract 2

Supplemental Digital Content is available in the text.

**Clinical Perspective**

**What Is New?**

- Once total cholesterol and high-density lipoprotein cholesterol are included in a primary cardiovascular disease risk prediction score in a cohort at low risk, there is no evidence that addition of other lipids or lipoproteins (apolipoprotein B, apolipoprotein A1, direct or calculated low-density lipoprotein cholesterol) appreciably improve prediction.
- Similar findings were reproduced in those taking a statin at baseline.

**What Are the Clinical Implications?**

- Calls for widespread use of apolipoproteins are not warranted given the negligible difference in risk prediction beyond total cholesterol and high-density lipoprotein cholesterol.
- Direct low-density lipoprotein cholesterol is also not required for risk prediction; non–high-density lipoprotein cholesterol is a cheaper equivalent predictor of risk on and off statins, without requirement for a fasting sample.

**Editorial, see p 553**

Hyperlipidemia has been widely documented to be associated with higher cardiovascular disease (CVD) risk,^1^ and pharmacological reduction of circulating non–high-density lipoprotein cholesterol (HDL-C) is beneficial in primary and secondary CVD prevention.^2,3^ Widely-used clinical CVD risk calculators frequently include classical biochemistry measures of total cholesterol, HDL-C, or a combination of these.^4–7^

Total cholesterol is an estimate of the cholesterol in all very-low-density lipoproteins, intermediate-density lipoproteins, low-density lipoproteins (LDL), high-density lipoprotein, and lipoprotein(a) in the fasted state, as well as chylomicrons and their remnant particles in the unfasted state. Notably, the majority of total cholesterol resides in LDL particles, and the additional clinical utility of recently developed direct LDL-cholesterol (LDL-C) measurements are not entirely clear. Non–HDL-C has been suggested as a pragmatic and cost-effective alternative to direct LDL-C measurement, requiring only total cholesterol and HDL-C to be performed.^8^

Apolipoproteins (Apo) A1 and B are the principle protein components of HDL and non-HDL particles, respectively, and have also been of interest for their potential in improving CVD risk prediction.^9,10^ This is partly because of the proteins being key components of lipoprotein function, and their measurement facilitating quantification of objective particle numbers.^8^ ApoB is thought to be of similar use in CVD prediction as non–HDL-C and LDL-C, but in some studies has shown improvements in prediction in scenarios where treated LDL-C levels are within the normal range.^11^ However, an Emerging Risk Factor Collaboration individual participant meta-analysis from 37 prospective cohorts (165 544 participants) suggested that replacement of traditional lipid parameters with ApoA1 and ApoB measurements only offered very modest improvement in CVD prediction.^12,13^ Despite this, enthusiasm for clinical use of apolipoprotein measurements beyond LDL-C or total cholesterol remains high in many places.^14^

Therefore, despite considerable research, identification of the most informative measures of blood lipids in terms of CVD risk is still a matter of some contention. UK Biobank is a large, prospective, population-based cohort study that has recently released data on baseline biochemistry measurements including routine lipids and apolipoproteins in participants who consented to blood sample collection. Participants were generally of an age range where CVD risk scoring is conducted in clinical practice. All blood tests were conducted in a single dedicated ISO17025 accredited biochemistry laboratory using robust methodologies. These data therefore might be more robust than data from some constituent studies in previous meta-analyses and may robustly address controversies around biomarker utility in risk scoring. The aim of this study was to investigate whether additional lipid/lipoprotein measurements improve prediction of CVD events over established risk scores.

## Methods

The data reported in this article are available via application to the UK Biobank to other researchers for purposes of reproducing the results or replicating the procedure.

UK Biobank recruited 502 639 participants (aged 37–73 years) from 22 assessment centers across the UK between 2007 and 2010. Baseline biological measurements were recorded, and touch-screen questionnaires were administered, as described elsewhere.^15,16^ UK Biobank received ethical approval from the North West Multi-Center Research Ethics Committee (11/NW/03820). All participants gave written informed consent before enrollment in the study, which was conducted in accordance with the principles of the Declaration of Helsinki.

The first (of 2) measurements of systolic and diastolic blood pressure were used for these analyses, preferentially using an automated measurement. Repeated blood pressure measurements were very similar and highly correlated.^17^ Smoking status was categorized into never or former/current smoking for this analysis. Ethnicity was coded as white, black, South Asian, or other. Nonfasting venous blood sampling was conducted, and collection procedures for the study were validated.^18^ Biochemistry measures were performed at a dedicated central laboratory between 2014 and 2017, including serum total cholesterol, HDL-C, triglycerides, direct LDL-C, and ApoA1 and ApoB. Non–HDL-C was calculated as total minus HDL-C.^19^ LDL-C was additionally calculated using 2 formulae/schema: Friedewald and Martin/Hopkins. Friedewald LDL is not calculated in 10 884 patients with triglycerides >400 mg/dL (2.2%). In this group, a direct LDL measurement was used as a substitute measure to minimize loss of data and mimics a potential clinical scenario where patients with a high triglycerides result may automatically have LDL directly measured. Remnant cholesterol was also calculated (Methods in the online-only Data Supplement). Further details of these measurements and assay performances can be found in the UK Biobank online showcase and protocol,^20^ and further information is listed in the supplement (Methods in the online-only Data Supplement).

The definition of known baseline diabetes included self-reported type 1 or type 2 diabetes (either as a stand-alone reported illness, or in response to a specific question about doctor-diagnosed diabetes) or those who reported using insulin. Statin and antihypertensive medication use were self-reported. Baseline cardiovascular disease was defined as self-reported historic angina, myocardial infarction, stroke, or transient ischemic attack.

Date and cause of death were obtained from death certificates held by the National Health Service Information Center for participants from England and Wales and the National Health Service Central Register Scotland for participants from Scotland. There were 2 outcomes of interest in the current study. The primary outcome was incident fatal and nonfatal cardiovascular disease that reflects the American College of Cardiology/American Heart Association guidelines prediction score including death from cardiovascular disease as the underlying cause (*International Classification of Diseases, Tenth Revision*:[*ICD-10*]:I20–25, I60–64) or hospitalization for cardiovascular disease (*ICD-10*:I21, I22, I60–64)^7^ from Hospital Episode Statistics. A secondary outcome was death from cardiovascular disease as the underlying cause specifically (*ICD-10*:I10–15, I44–51, I20–25, I61–73) (Methods in the online-only Data Supplement), reflecting outcomes used in the European Systematic Coronary Risk Evaluation (SCORE) clinical guidelines.^21^

The period at risk per participant began on the date of their assessment. End of follow-up for each participant was recorded as the date of death or the date of end of follow-up for the assessment center attended (January 31, 2018, for assessment centers in Wales or England and November 30, 2016, for participants in Scotland), or the first date of hospitalization for CVD (in outcome 1), whichever came first.

Participants with a baseline history of CVD or on statins were excluded from the primary analyses, but associations of lipids with CVD events were analyzed in this group as a secondary aim. All analyses were performed on those with complete data for exposures and covariates of interest.

### Statistical Analyses

Classical risk factors were expressed as mean (SD) as all were approximately normally distributed, and number (%) for categorical variables. Each lipid/protein variable was also categorized into quintiles. The distribution of classical risk factors by categories of each lipid/protein variable (total cholesterol, HDL-C, LDL-C, non–HDL-C, ApoA1, ApoB) were assessed using tests for trends across the variable using ANOVA, or a chi-squared test, as appropriate. Associations between classical risk factors and lipid/protein variables with CVD events were also tabulated using these methods.

Correlations between log-transformed total cholesterol, HDL-C, LDL-C, non–HDL-C, ApoA1, and ApoB were assessed using Pearson correlation coefficient, both without adjustment, then accounting for sex and age.

Event-free survival was initially explored for each quintile of lipid/protein using univariable Kaplan-Meier models for both incident and fatal CVD. The association of each lipid/protein variable with incident nonfatal and fatal CVD was tested using Cox proportional hazard models. Models for the incident fatal/nonfatal CVD event were adjusted for nonlipid variables broadly comparable to those used in the American College of Cardiology/American Heart Association guidelines: age, sex, ethnicity, systolic blood pressure, diastolic blood pressure, antihypertensive medication, diabetes, and smoking.^7^ Models of fatal CVD were adjusted for nonlipid variables used in the European SCORE clinical guidelines: age, sex, systolic blood pressure, and smoking.^21^ Cox proportional hazards models conducted in people who had baseline CVD or were taking a statin were also additionally adjusted for baseline CVD and statin use. Follow-up time was calculated as days from assessment to first CVD hospital diagnosis or CVD death, the end of follow-up, or loss to follow-up, whichever occurred first. We made qualitative comparisons between the hazard ratios (HR) for the lipid and lipoprotein variables; fixing the β coefficients for covariates in the models made no substantial difference to overall conclusions.

To visualize the relationship between each lipid/protein variable and outcome, restricted cubic splines with 4 knots (at the 5th, 35th, 65th, and 95th centiles^22^) were constructed for each lipid/protein variable, and the fully adjusted relationship (as above) with CVD events was plotted. Changing knots did not substantially change the shape of the associations (data not shown).

To assess the differential predictive ability of each lipid/protein, both alone and in combination, adjusted Cox proportional hazard models were constructed for each outcome (the lipid/protein variable was entered linearly into the model, or in the case of fatal CVD, lipid/protein variables were added as quintiles because of nonlinearity). Potential pairwise interactions between classical risk factors and lipid/protein variables were considered. The proportional hazard assumption was checked by visual inspection of Schöenfeld residuals. Utility of lipids to discriminate composite and fatal CVD events were then tested using Harrell C-statistics using an either/or model (testing whether a priori measurement of total and HDL-C, or ApoB and ApoA1, is preferable) or an additive model (whether other lipids and lipoproteins add to total and HDL-C). We also investigated model fit using changes in Akaike Information Criteria and Bayesian Information Criteria. We used a categorical net reclassification index, with 1000 bootstraps to estimate 95% CI, to investigate the changes in predicted risk classification across the 7.5% 10-year risk boundary used to allocate statins in the American College of Cardiology/American Heart Association model.^23^

A discordance sensitivity analysis was also performed to check utility of LDL-C and apolipoprotein measurement in a group where ApoB and direct LDL-C were not in “agreement.” The absolute difference in the percentile of ApoB and direct LDL for each participant was used to stratify the population into a discordant group (those with an absolute difference of >10 percentage points between their ApoB and LDL percentiles)^24^ where utility of lipid and lipoprotein measurements were checked.

All analyses were performed using STATA 14 (StataCorp LP) and R3.5.1 (including package nricens^25^). A *P* value of <0.05 was considered statistically significant.

## Results

### Baseline Results

Of 413 326 participants with no history of CVD or statin use, complete data on covariates of interest, including lipid/protein variables, were available in 346 686 participants (83.9%). Data were also available in 68 649 participants taking a statin with or without baseline CVD (secondary analysis).

In those with no history of CVD and not taking a statin, higher total cholesterol, ApoB, direct LDL-C, and non–HDL-C were all associated with older age, and higher systolic and diastolic blood pressure (Tables I through IV in the online-only Data Supplement). Women were more likely to have high total cholesterol levels (Table I in the online-only Data Supplement), whereas men were more likely to have high ApoB levels (Table II in the online-only Data Supplement). Both HDL-C and ApoA1 were associated with older age and were higher in women (Tables V and VI in the online-only Data Supplement). ApoB, direct and calculated LDL-C, and non–HDL-C were all highly correlated with each other (*r*>0.90), while HDL-C was strongly associated with ApoA1 (*r*=0.924) (Table 1).

**Table 1.**
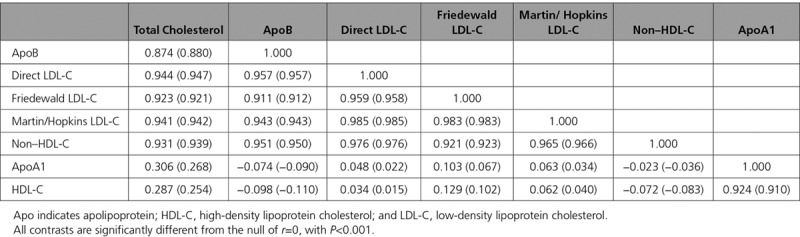
Pearson Correlation Coefficients *r* (and Age- and Sex-Adjusted *r*) Between Each Pair of Lipids or Proteins in Those Without Baseline Cardiovascular Disease and Not Taking a Statin (n=346 686)

### Univariable Associations of ApoB and ApoA1 with CVD Outcomes

Median follow up time was 8.9 years (Q1–Q3: 8.2–9.5). The incident fatal/nonfatal CVD outcome occurred in 6216 participants (1.8%), and fatal CVD occurred in 1656 participants (0.5%).

Incident CVD events occurred more frequently in those participants with more adverse risk profiles, including more frequently being older, male, smokers, and with higher systolic blood pressure, diastolic blood pressure, total cholesterol, lower HDL-C and ApoA1, and higher LDL-C and ApoB (Table 2). Similar patterns were seen for the fatal CVD events (Table VII in the online-only Data Supplement). In Kaplan-Meier curves, lipids and lipoproteins by quintiles had strong univariable associations with the incident CVD event (Figure I in the online-only Data Supplement). Similar associations were seen for fatal CVD, although the separation of the survival curves for total cholesterol was less clear (Figure II in the online-only Data Supplement).

**Table 2.**
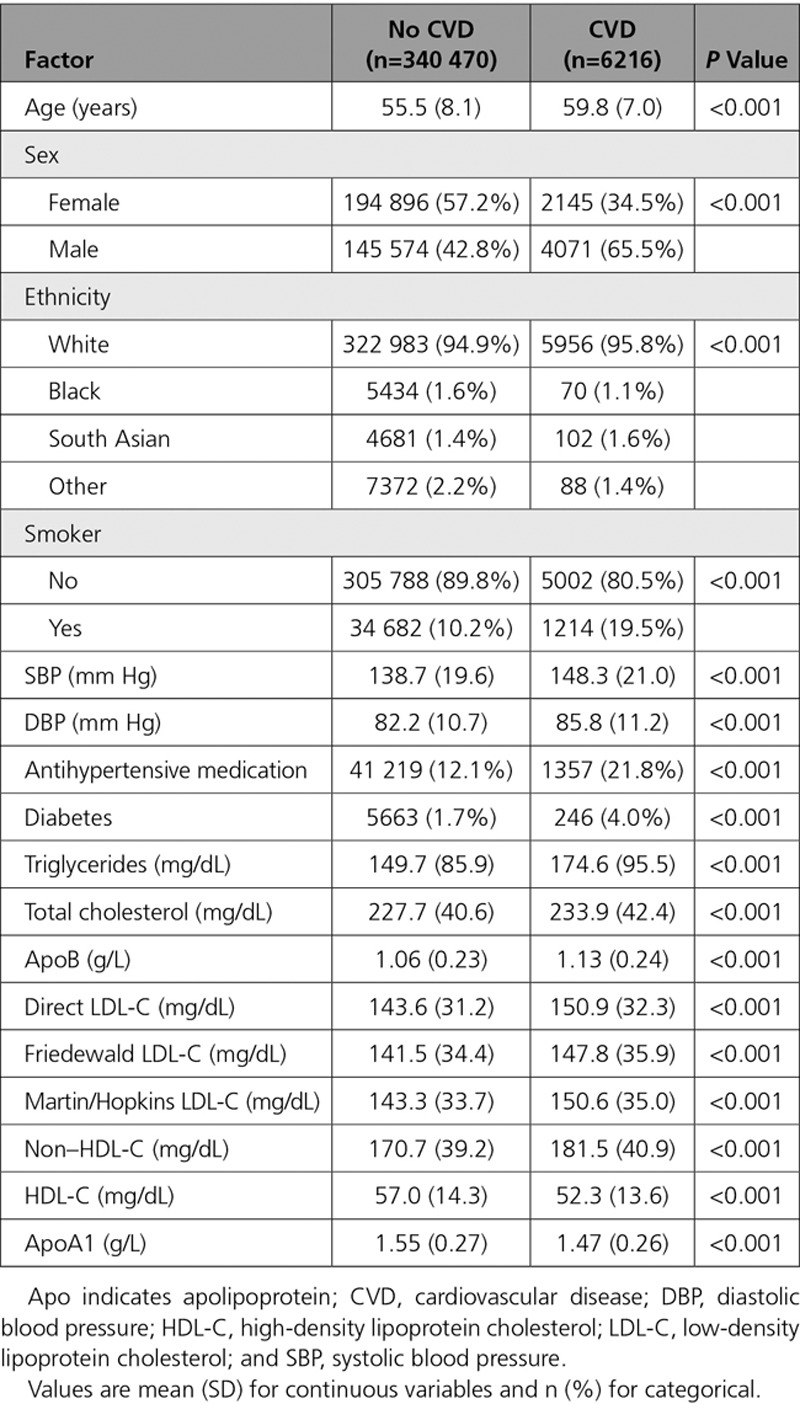
Distribution of Baseline Characteristics in UK Biobank Participants by Incident Fatal/Nonfatal CVD Status in Those Without Baseline CVD and Not Taking a Statin (n=346 686)

### Multivariable Association of ApoA1 and ApoB with Outcomes

**Figure 1.**
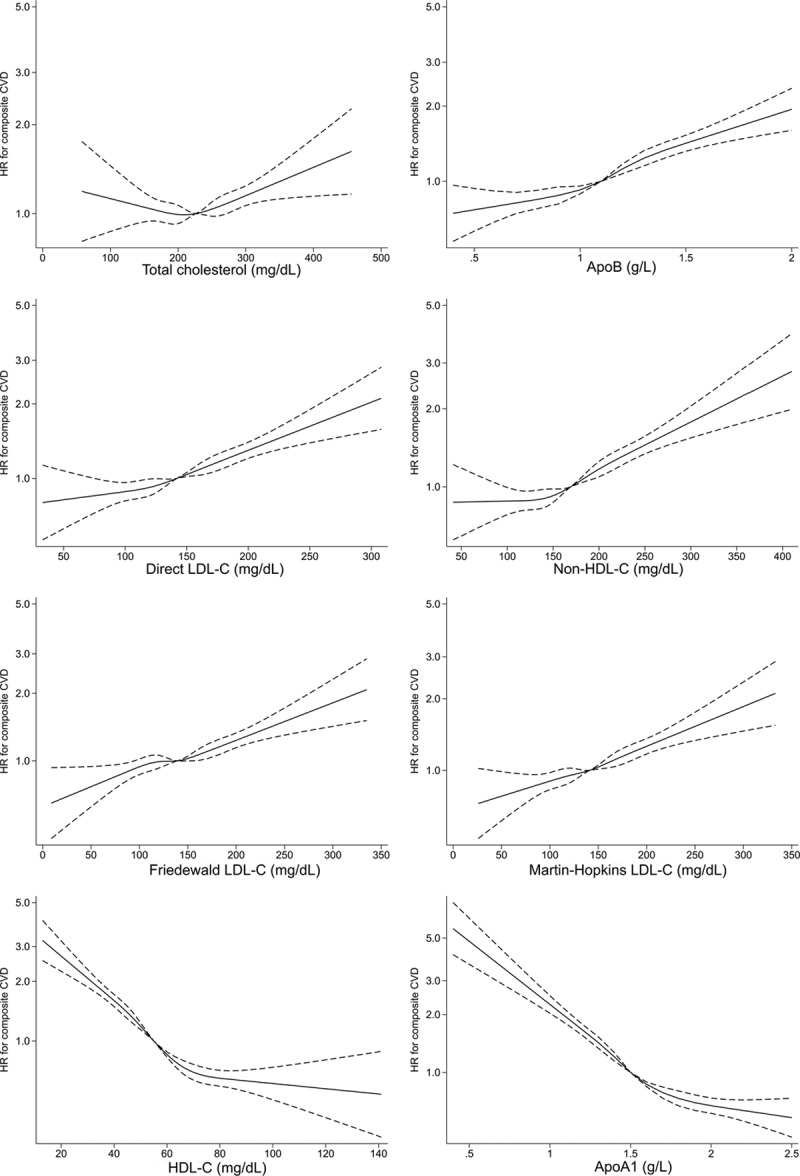
**Adjusted association of total cholesterol, ApoB, direct LDL-C, non–HDL-C, calculated LDL-C, HDL-C, and ApoA1 with composite fatal/nonfatal CVD events in those without baseline CVD and not taking a statin.** Apo indicates apolipoprotein; CVD, cardiovascular disease; HDL-, high-density lipoprotein cholesterol; HR, hazard ratio; and LDL, low-density lipoprotein cholesterol.

In Cox models adjusting for nonlipid classical risk factors, the shapes of the association of total cholesterol, non–HDL-C, and ApoB with risk (HR) of the composite CVD outcome were positive, with increases in the gradient of risk at around 220 mg/dL for total cholesterol and 1.1g/L for ApoB (Figure 1). The shape of the association of HDL-C and ApoA1 with the risk of CVD was inverse (Figure 1). HDL-C and ApoA1 had the strongest (inverse) associations with risk of fatal CVD (Figure III in the online-only Data Supplement), and the shape of the association of total cholesterol with risk of fatal CVD was “U-shaped” (Figure III in the online-only Data Supplement). Exclusion of the first 2 years of follow-up did not substantially attenuate these results (Figure IV in the online-only Data Supplement).

After adjustment for classical risk factors, 1 SD increase in ApoB (0.23 g/L), LDL-C (31.2 mg/dL), and non–HDL-C (39.2 mg/dL) had similar associations with incident CVD events (HR, 1.23, 1.20, and 1.21, respectively), as did measures of calculated LDL-C (Tables 3 and 4). Associations for 1 SD increase in HDL-C (14.3 mg/dL) and ApoA1 (0.27g/L) were also similar (HR, 0.81 [both]) (Tables 3 and 4). Patterns were similar for those on statins (Tables VIII and IX in the online-only Data Supplement). Specifically, 1 SD increments in ApoB, LDL-C, and non–HDL-C were broadly equally associated with subsequent CVD risk (HR, 1.16, 1.15, and 1.16, respectively). In the discordance sensitivity analysis, ApoB had stronger associations with CVD than LDL-C and non–HDL-C (Tables X and XI in the online-only Data Supplement). There was evidence that ApoB and LDL-C measurements were more strongly associated with risk in men, at younger age, in people who smoke, and in those with high triglycerides, although the association was still positive in all subgroups (Table XII in the online-only Data Supplement). Generally, interactions were less pronounced for HDL-C and ApoA1 (Table XII in the online-only Data Supplement).

**Table 3.**
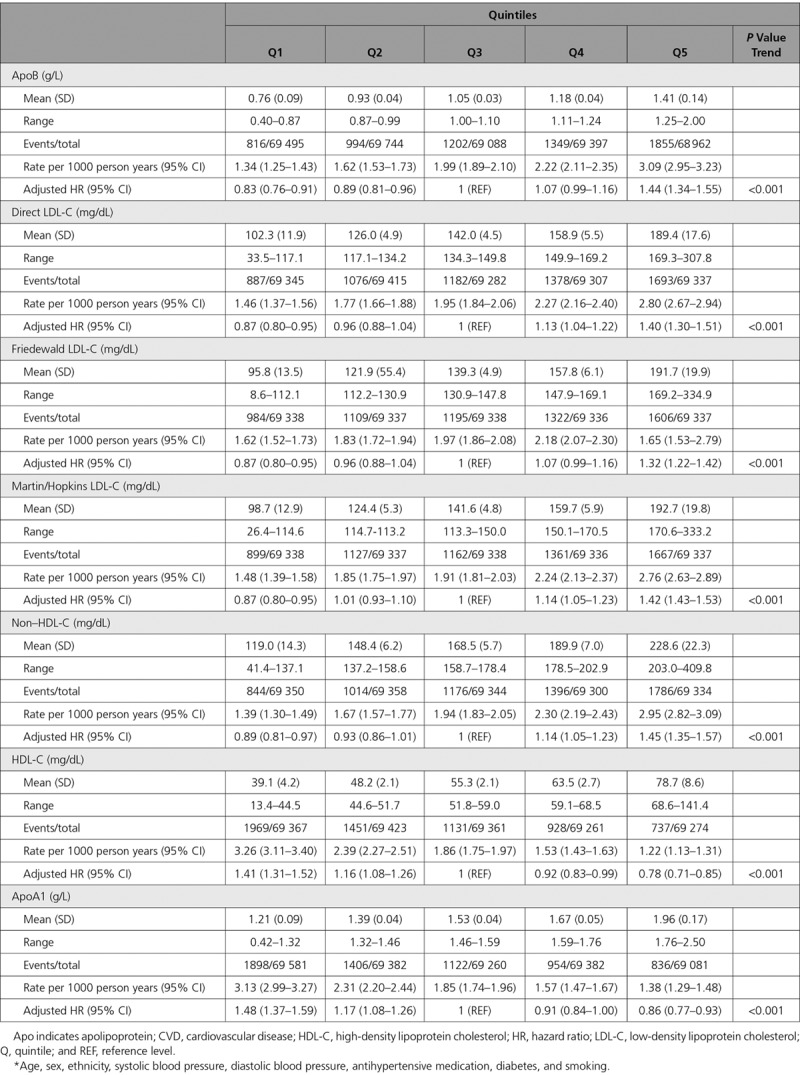
Adjusted* Associations of Different Lipid and Lipoprotein Measures with Composite Cardiovascular Disease, by Quintile of the Distribution in Those Without Baseline CVD and Not Taking a Statin (n=346 686)

**Table 4.**
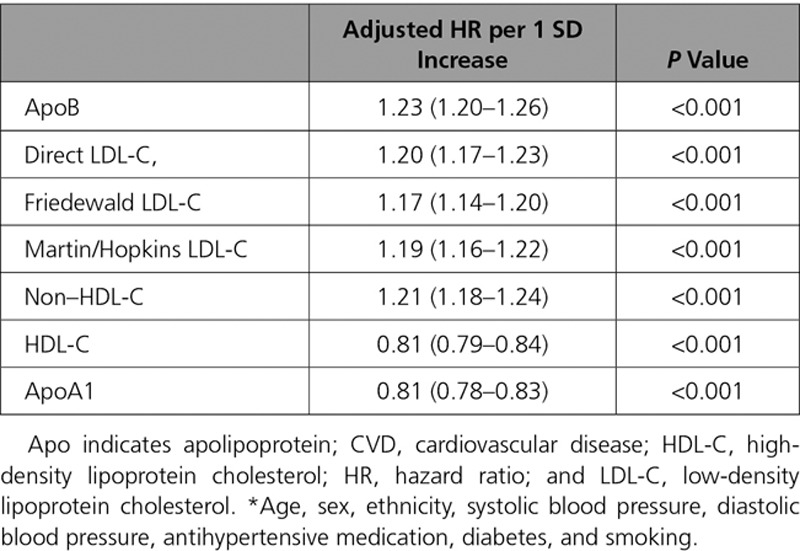
Adjusted* Associations of Different Lipid and Lipoprotein Measures With Composite Cardiovascular Disease Using a Linear Model in Those Without Baseline CVD and Not Taking a Statin (n=346 686)

### Prediction of CVD Events in Addition to Classical Risk Factors

**Figure 2.**
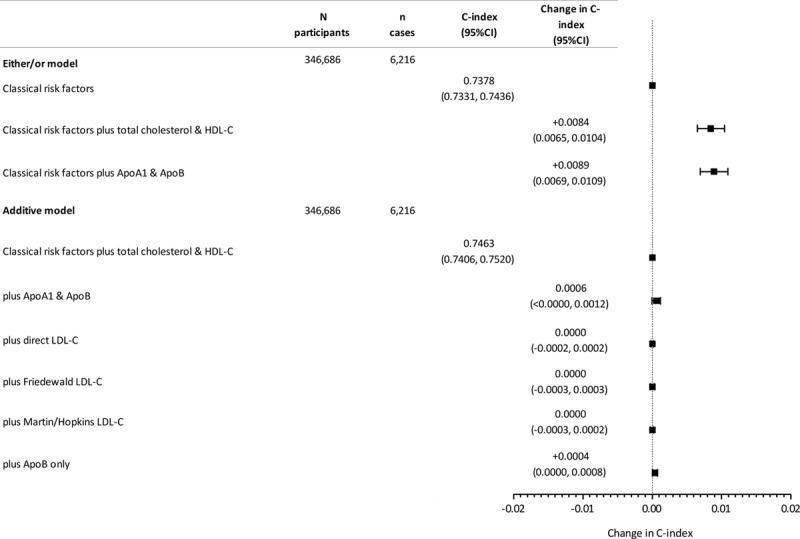
**Change in C-index for prediction of incident fatal/nonfatal CVD on addition or substitution of lipids and apolipoproteins to classical CVD risk factors in those with no baseline CVD and not taking a statin.** Apo indicates apolipoprotein; CVD, cardiovascular disease; HDL, high-density lipoprotein cholesterol; and LDL, low-density lipoprotein cholesterol.

In a model of CVD prediction, classical risk factors without lipids yielded reasonable discrimination (C-index, 0.7378; 95% CI, 0.7331, 0.7436) (Figure 2). Discrimination was improved on the addition of total cholesterol and HDL-C (C-index change, 0.0084; 95% CI, 0.0065, 0.0104). There was near identical improvement on addition of ApoA1 and ApoB instead of total cholesterol and HDL-C (C-index change, 0.0089; 95% CI, 0.0069, 0.0109) (Figure 2). However, adding ApoB plus ApoA1 to total cholesterol and HDL did not improve discrimination (C-index change, 0.0006; 95% CI, −0.00003, 0.0012) (Figure 2). Adding any measure of LDL-C or ApoB alone to a model already containing total and HDL-C also offered no substantial discriminative benefit (Figure 2). Remnant cholesterol also did not add discrimination once total and HDL-cholesterol were in the model (C-index change, −0.0001; 95% CI, −0.0003, 0.0003). Non–HDL-C was not added to the model, attributable to collinearity with a model already containing total and HDL-C. There was also no strong evidence of improvement in goodness of model fit on addition of additional lipids and lipoproteins as measured by Akaike Information Criteria and Bayesian Information Criteria (Table XIII in the online-only Data Supplement). In analysis of the categorical net reclassification index across the 7.5% 10-year risk threshold, there was some evidence that addition of either total cholesterol and HDL-C or ApoB and ApoA1 improved classification, particularly among cases (Table XIV in the online-only Data Supplement). However once total cholesterol and HDL-C were in the model, addition of any measurement of LDL-C or apolipoprotein did not improve classification among cases or controls, and the upper end of the 95% CI did not exceed 0.51% improvement in any overall model (Table XIV in the online-only Data Supplement).

Similarly, in a model of fatal CVD prediction based on European SCORE risk factors plus current treatments, classical risk factors without lipids yielded a C-index of 0.7813 in the cohort without baseline CVD and not taking a statin (95% CI, 0.7708, 0.7919). Discrimination was improved on the addition of total and HDL-C (C-index change, 0.0036; 95% CI, 0.0015, 0.0056). There was a similar improvement on addition of ApoA1 and ApoB (C-index change, 0.0045; 95% CI, 0.0021, 0.0069) (Figure V in the online-only Data Supplement). Adding other lipids or proteins to a model already containing total and HDL-C generally offered no discriminative benefit for fatal CVD (Figure V in the online-only Data Supplement).

In the discordance sensitivity analysis, classical risk factors with total cholesterol and HDL-C yielded a C-index of 0.7444 of the fatal/nonfatal CVD outcome (95% CI, 0.7317, 0.7572), which was not improved on the addition of ApoA1 and ApoB (change 0.0007; 95% CI, −0.0011, 0.0024), direct LDL (−0.0001; 95% CI, −0.0004, 0.0002), Friedewald LDL-C (−0.0001; 95% CI, −0.0010, 0.0009), or Martins-Hopkins LDL (−0.0002; 95% CI, −0.0008, 0.0005).

In participants taking a statin, classical risk factors (including baseline CVD status) also yielded reasonable discrimination of the fatal/nonfatal CVD outcome (C-index, 0.7118; 95% CI, 0.7033, 0.7203). Adding non-HDL improved discrimination (C-index change, 0.0030; 95% CI, 0.0012, 0.0048), as did adding ApoB or any lipid measure (Figure VI in the online-only Data Supplement). However, adding ApoB or LDL to a model already containing non-HDL did not further improve discrimination (Figure VI in the online-only Data Supplement).

## Discussion

In this large and comprehensive single cohort, we clearly demonstrate that total cholesterol and HDL-C, even when measured in the nonfasting state, adequately capture conventional lipid-associated cardiovascular risk for primary CVD outcomes. Apolipoproteins give negligible additional predictive value when they either replace total cholesterol and HDL-C or are added on top of them. We also show that for people on statins, ApoB, direct and measured LDL-C, and non–HDL-C have very similar associations with incident CVD, and addition of further lipid measurements does not substantially improve non–HDL-C in terms of prediction. We also show that while all lipids are broadly linearly related to incident fatal/nonfatal CVD outcomes, the relationship of lipids and lipoproteins to fatal CVD, as used in SCORE, is often nonlinear, which may complicate risk prediction for scores using only fatal outcomes. Given that all assays in UK Biobank were conducted in a single center using robust routine assays, these results from over 400 000 participants add considerable evidence to the literature. They suggest there is no need for widespread measurement of the much more costly apolipoproteins or direct LDL-C in usual risk factor screening for CVD when cheaper measures of total cholesterol and HDL-C are available.

There has been considerable research suggesting apolipoproteins may improve CVD risk over measurement of total cholesterol and HDL-C. The INTERHEART study used apolipoproteins and showed them to be strongly predictive of risk; however, this was a case-control study, which has well-established limitations, and the study did not directly compare lipids and apolipoproteins incremental discrimination on addition to a risk score containing classical risk factors.^26^ Individual participant meta-analysis suggested that replacement of traditional lipid parameters with ApoA1 and ApoB measurements only offered modest improvement in CVD prediction.^13,27^ The limitations of that study included use of different assays in constituent studies, increasing the possibility of exposure misclassification. Indeed, despite the Emerging Risk Factor Collaboration results, many investigators and guidelines have continued to recommend measurement of apolipoproteins in risk factor assessment.^9,14,28,29^ Our results broadly fit and extend data from Emerging Risk Factor Collaboration and are in line with recommendations made by other investigators,^30^ as well as recent clinical guidelines; indeed, in these guidelines, it was noted the apolipoproteins were not available to all clinicians and were more expensive than usual lipid measures.^4^ Furthermore, a recent study showed that ApoB may be more informative than LDL-C in estimating the impact of genetic variation in lipid processing pathways.^31^ These data might lead to a reinvigoration of arguments for measuring ApoB in risk prediction. However, such data cannot be extrapolated to the risk prediction environment in people in the general population being considered for lipid lowering therapy.

We acknowledge that risk prediction metrics in the current study do not necessarily inform us about the clinical implications of reductions in different lipid parameters on treatment.^32^ However, the use of non–HDL-C has been suggested as a potential treatment target of lipid-lowering therapy in a meta-analysis of statin trials.^33^ In the present UK Biobank study, non–HDL-C was as strongly associated with incident CVD as ApoB and LDL-C for patients on statins, with HRs for 1 SD increase being almost identical. The fact that non–HDL-C can be performed using nonfasting samples, and is cheaper to measure, means this should be the default measurement to inform treatment decisions in those on statins. However, we accept further work is required to raise awareness of non–HDL-C in the clinical community and to validate it as a treatment target.^34^ In the discordance analysis, ApoB was more strongly associated with CVD than LDL-C or non–HDL-C, but even in this highly selected subset (18% of the main cohort), there was no improvement in discrimination when apolipoproteins were added to the risk score model.

The strengths of our study include the large cohort size at an age relevant to CVD risk scoring, biochemistry assays performed consistent with the internationally recognized standard for testing and calibration laboratories, and using models with comprehensive adjustment for all usual cardiovascular risk factors. We were also able to separately analyze participants already on statins as well as those with previous CVD. The numbers of events accrued in the study are also considerable in the context of existing literature. Passive collection of Hospital Episode Statistics data has the advantage over active cohort studies that loss to follow-up is minimized. The primary reason for loss to follow-up here is emigration. Estimates suggest that emigration in the UK Biobank cohort is minimal (≈0.3%).^35^ The use of Hospital Episode Statistics data to identify hospitalization for CVD shows reasonable accuracy compared to active biomedical examinations; for instance, the sensitivity and specificity for coronary heart disease have been reported at 72% and 96%.^36,37^ Further potential limitations include the relatively low average CVD risk of participants. Even so, baseline risk factors were predictive of CVD in the expected manner, and risk prediction models performed broadly in line with expectations. We were unable to update participant risk during follow-up for new initiation of statin therapy, although we did consider those taking statins as a separate group and report similar findings, suggesting the influence of statins and changes in statin status during follow-up are likely to be minimal. Separate studies would be required to investigate the utility of different measures of cholesterol and apolipoproteins in investigating statin efficacy on CVD events.^38^ UK Biobank is not representative of the whole UK population, but this is generally not a concern in investigating risk associations.^39^ Incident events were not adjudicated, as they would be in a clinical trial, but ascertainment of such events has been in line with other cohort studies used to generate risk scores.^40,41^ The C-statistic has been criticized for a lack of sensitivity to potentially important clinical changes in predictive utility, but the results of our analysis using the net reclassification index are consistent with increments in the C-statistic. Finally, as most of the participants were white, separate work is needed to validate these findings in different races.

In conclusion, data from UK Biobank show that the predictive ability of total cholesterol and HDL-C, in the context of other classical risk factors, are not improved by the addition or replacement with apolipoproteins in the assessment of CVD risk. Similarly, non–HDL-C appears adequate to assess on-treatment lipid lowering therapy in this setting. Although lipoprotein(a) measurements may have a role in some settings, these large scale data support the measurement of standard total cholesterol and HDL-C in the nonfasting setting as being perfectly adequate in their capture of lipid-associated CVD risk and for determining non–HDL-C as a treatment target for those already on statins.

## Acknowledgments

This research was conducted using the UK Biobank resource. We thank the participants of the UK Biobank. The work was performed under UK Biobank project number 9310.

## Sources of Funding

The work in this study was supported by a grant from Chest, Heart, and Stroke Association Scotland [Res16/A165]. R. Brown was supported by a Medical Research Council Doctoral Training Partnership MR/N013166/1.

## Disclosures

Dr Paul Welsh has received grant support from Roche, AstraZeneca, and Boehringer Ingelheim. Dr Sattar has received fees for consulting, speaking, and/or honoraria from Roche Diagnostics, AstraZeneca, Boehringer Ingelheim, NovoNordisk, Amgen, Sanofi/Regeneron, and Janssen.

## Supplementary Material
