# Supplementary material for: Comparison of Conventional Lipoprotein Tests and Apolipoproteins in the Prediction of Cardiovascular Disease: Data From UK Biobank
Source: Circulation. 2019 Jun 20;140(7):542–52. doi: 10.1161/CIRCULATIONAHA.119.041149 (PMC6693929; doi:10.1161/CIRCULATIONAHA.119.041149)
Supplement: Supplementary file 1 [file cir-140-542-s001.pdf]

SUPPLEMENTAL MATERIAL

Welsh et al

Comparison of conventional lipoprotein tests and apolipoproteins in the prediction of cardiovascular disease: data from UK Biobank

Supplementary methods

Biochemical measurements

Blood collection procedures were dictated by a standard operating procedure for the UK Biobank study [1]. Blood collection sampling procedures for the study were validated [2]. Non fasting serum samples were collected by a trained phlebotomist. Refrigerators used for storing the blood, urine and saliva samples were kept between 2 to 8°C. Samples were transferred to a central laboratory for storage on a daily basis.

Blood samples were transferred to a dedicated central laboratory between 2014 and 2017 for archiving at -80°C until analysis using a standard operating procedure [3]. Serum samples were centrifuged for 10 minutes at 2000 RCF. Biochemistry measures were performed on automated analysers, including serum total cholesterol and HDL-C (Beckman Coulter AU5400), direct LDL-C (Beckman Coulter AU5400), and ApoA1 and ApoB (Beckman Coulter AU5800) [4]. Three levels of quality control were used for each assay. Across these, coefficients of variation (CV) for each of ApoA1, ApoB, and triglycerides were <3% and for total cholesterol, HDL-C and LDL-C were <2%. Each assay was registered with an external quality assurance (EQA) scheme, and assay performance was externally verified via the results returned from participation in these schemes. Data were adjusted by UK Biobank centrally before release to adjust for pre-analytical variables [5].

Friedewald and Martin/Hopkins method for deriving calculated LDL were used as per published methods [6]. Remnant cholesterol was calculated as Total cholesterol - (Friedewald LDL-c+HDL-C).

Outcome definitions

ICD-10 codes for CVD events included:

<u>Composite nonfatal/fatal CVD events (representing AHA/ACC CVD definition) –</u>

Fatal codes

I20 - Angina pectoris

I21 - Acute myocardial infarction

I22 - Subsequent MI

I23 - Certain current complications following acute myocardial infarction

I24 - Other acute ischaemic heart diseases

I25 - Chronic ischaemic heart disease

I60 - Subarachnoid haemorrhage

I61 - Intracerebral haemorrhage

I62 - Other nontraumatic intracranial haemorrhage

I63 - Cerebral infarction

I64 - Stroke, not specified as haemorrhage or infarction

Non-Fatal codes

I21 - Acute myocardial infarction

I22 - Subsequent MI

I60 - Subarachnoid haemorrhage

I61 - Intracerebral haemorrhage

I62 - Other nontraumatic intracranial haemorrhage

I63 - Cerebral infarction

I64 - Stroke, not specified as haemorrhage or infarction

<u>Fatal CVD (representing SCORE CVD definition) -</u>

I10 - Essential (primary) hypertension

I11 - Hypertensive heart disease

I12 - Hypertensive renal disease

I13 - Hypertensive heart and renal disease

I15 - Secondary hypertension

I20 - Angina pectoris

I21 - Acute myocardial infarction

I22 - Subsequent MI

I23 - Certain current complications following acute myocardial infarction

I24 - Other acute ischaemic heart diseases

I25 - Chronic ischaemic heart disease

I44 - Atrioventricular and left bundle-branch block

I45 - Other conduction disorders

I46 - Cardiac arrest

I47 - Paroxysmal tachycardia

I48 - Atrial fibrillation and flutter

I49 - Other cardiac arrhythmias

I50 - Heart failure

I51 - Complications and ill-defined descriptions of heart disease

I61 - Intracerebral haemorrhage

I62 - Other nontraumatic intracranial haemorrhage

I63 - Cerebral infarction

I64 - Stroke, not specified as haemorrhage or infarction

I65 - Occlusion and stenosis of precerebral arteries, not resulting in cerebral infarction

I66 - Occlusion and stenosis of cerebral arteries, not resulting in cerebral infarction

I67 - Other cerebrovascular diseases

I68 - Cerebrovascular disorders in diseases classified elsewhere

I69 - Sequelae of cerebrovascular disease

I70 - Atherosclerosis

I71 - Aortic aneurysm and dissection

I72 - Other aneurysm and dissection

I73 - Other peripheral vascular diseases

Supplemental Table 1. Baseline characteristics across quintiles of total cholesterol among 346,686 UK Biobank participants.

Factor		Total cholesterol mg/dL quintiles, mean (SD)					P-value
		Q1: 173.84 (16.13) n=69415	Q2: 205.06 (6.33) n=69344	Q3: 225.71 (5.79) n=69280	Q4: 247.49 (7.14) n=69398	Q5: 287.21 (23.15) n=69249	
Age	years	52.69 (8.47)	54.55 (8.20)	55.79 (7.93)	56.80 (7.58)	57.82 (7.17)	<0.001
Sex	Female	35246 (50.8%)	36994 (53.3%)	38542 (55.6%)	40931 (59.0%)	45328 (65.5%)	<0.001
	Male	34169 (49.2%)	32350 (46.7%)	30738 (44.4%)	28467 (41.0%)	23921 (34.5%)	
Ethnicity	White	63943 (92.1%)	65349 (94.2%)	65920 (95.2%)	66668 (96.1%)	67059 (96.8%)	<0.001
	Black	2145 (3.1%)	1202 (1.7%)	912 (1.3%)	730 (1.1%)	515 (0.7%)	
	South Asian	1357 (2.0%)	1156 (1.7%)	970 (1.4%)	739 (1.1%)	561 (0.8%)	
	Other	1970 (2.8%)	1637 (2.4%)	1478 (2.1%)	1261 (1.8%)	1114 (1.6%)	
Smoking status	Never	61852 (89.1%)	62251 (89.8%)	62254 (89.9%)	62451 (90.0%)	61982 (89.5%)	<0.001
	Ever	7563 (10.9%)	7093 (10.2%)	7026 (10.1%)	6947 (10.0%)	7267 (10.5%)	
SBP	mmHg	134.16 (19.00)	137.13 (19.26)	139.12 (19.30)	140.91 (19.48)	143.18 (19.91)	<0.001
DBP	mmHg	80.21 (10.73)	81.54 (10.67)	82.46 (10.60)	83.19 (10.60)	84.14 (10.63)	<0.001
Antihypertensive medication		9027 (13.0%)	8589 (12.4%)	8572 (12.4%)	8580 (12.4%)	7808 (11.3%)	<0.001
Diabetes mellitus		2864 (4.1%)	1236 (1.8%)	768 (1.1%)	576 (0.8%)	465 (0.7%)	<0.001
Triglyceride	mg/dL	118.03 (66.63)	134.78 (73.94)	147.41 (79.76)	161.93 (87.19)	188.84 (101.47)	<0.001
ApoB	g/L	0.80 (0.12)	0.95 (0.11)	1.05 (0.11)	1.16 (0.12)	1.37 (0.17)	<0.001
Direct LDL-C	mg/dL	104.70 (14.16)	126.96 (10.69)	141.97 (10.95)	157.92 (11.70)	187.17 (20.20)	<0.001

Friedewald LDL-C	mg/dL	99.56 (16.70)	123.35 (13.12)	139.53 (13.33)	156.76 (13.99)	188.88 (23.25)	<0.001
Martin/Hopkins LDL-C	mg/dL	101.42 (15.47)	125.34 (11.64)	141.58 (11.87)	158.68 (12.61)	190.24 (22.62)	<0.001
Non-HDL-C	mg/dL	122.83 (17.87)	149.82 (14.53)	168.36 (14.95)	188.24 (15.85)	225.30 (26.20)	<0.001
HDL-C	mg/dL	51.02 (12.27)	55.24 (13.38)	57.34 (14.01)	59.25 (14.41)	61.91 (14.71)	<0.001
ApoA1	g/L	1.43 (0.23)	1.52 (0.25)	1.56 (0.26)	1.60 (0.27)	1.65 (0.28)	<0.001

ApoA1 Apolipoprotein A1; ApoB Apolipoprotein B; CVD cardiovascular disease; DBP diastolic blood pressure; HDL high density lipoprotein; LDL low-density lipoprotein; SBP systolic blood pressure; Q quintile.

Supplemental Table 2. Baseline characteristics across quintiles of ApoB among 346,686 UK Biobank participants.

Factor		ApoB g/L quintiles: mean (SD)					P-value
		Q1: 0.76 (0.09) n=69495	Q2: 0.93 (0.04) n=69744	Q3: 1.05 (0.03) n=69088	Q4: 1.18 (0.04) n=69397	Q5: 1.41 (0.14) n=68962	
Age	years	52.83 (8.34)	54.88 (8.16)	56.05 (7.93)	56.70 (7.71)	57.20 (7.50)	<0.001
Sex	Female	42645 (61.4%)	40408 (57.9%)	38470 (55.7%)	37729 (54.4%)	37789 (54.8%)	<0.001
	Male	26850 (38.6%)	29336 (42.1%)	30618 (44.3%)	31668 (45.6%)	31173 (45.2%)	
Ethnicity	White	64920 (93.4%)	65888 (94.5%)	65581 (94.9%)	66312 (95.6%)	66238 (96.0%)	<0.001
	Black	1830 (2.6%)	1244 (1.8%)	947 (1.4%)	796 (1.1%)	687 (1.0%)	
	South Asian	968 (1.4%)	1042 (1.5%)	1083 (1.6%)	919 (1.3%)	771 (1.1%)	
	Other	1777 (2.6%)	1570 (2.3%)	1477 (2.1%)	1370 (2.0%)	1266 (1.8%)	
Smoking status	Never	62760 (90.3%)	63153 (90.5%)	62446 (90.4%)	61965 (89.3%)	60466 (87.7%)	<0.001
	Ever	6735 (9.7%)	6591 (9.5%)	6642 (9.6%)	7432 (10.7%)	8496 (12.3%)	
SBP	mmHg	133.59 (19.21)	137.11 (19.28)	139.30 (19.25)	141.03 (19.33)	143.52 (19.65)	<0.001
DBP	mmHg	79.52 (10.66)	81.31 (10.58)	82.40 (10.50)	83.47 (10.55)	84.85 (10.59)	<0.001
Antihypertensive medication		7598 (10.9%)	8566 (12.3%)	9055 (13.1%)	8986 (12.9%)	8371 (12.1%)	<0.001
Diabetes mellitus		2230 (3.2%)	1301 (1.9%)	995 (1.4%)	741 (1.1%)	642 (0.9%)	<0.001
Triglycerides	mg/dL	111.53 (68.09)	130.02 (73.06)	146.41 (79.53)	165.09 (84.85)	198.30 (95.65)	<0.001
Total cholesterol	mg/dL	180.88 (23.42)	207.32 (19.52)	225.55 (19.33)	245.10 (20.05)	280.82 (28.95)	<0.001

ApoB	g/L	0.76 (0.09)	0.93 (0.04)	1.05 (0.03)	1.18 (0.04)	1.41 (0.14)	<0.001
Direct LDL-C	mg/dL	103.87 (13.71)	126.69 (9.18)	142.28 (9.34)	158.69 (10.54)	187.51 (19.72)	<0.001
Friedewald LDL-C	mg/dL	99.53 (16.60)	123.85 (13.14)	140.21 (13.95)	157.27 (15.73)	187.56 (24.61)	<0.001
Martin/Hopkins LDL-C	mg/dL	100.81 (15.29)	125.53 (10.80)	142.14 (11.34)	159.49 (12.99)	189.64 (23.05)	<0.001
Non-HDL-C	mg/dL	121.42 (17.70)	149.36 (12.41)	168.79 (12.51)	189.44 (13.67)	225.94 (24.92)	<0.001
HDL-C	mg/dL	59.47 (15.45)	57.97 (14.74)	56.76 (14.25)	55.66 (13.66)	54.87 (12.61)	<0.001
ApoA1	g/L	1.58 (0.28)	1.57 (0.27)	1.55 (0.27)	1.54 (0.26)	1.52 (0.25)	<0.001

ApoA1 Apolipoprotein A1; ApoB Apolipoprotein B; CVD cardiovascular disease; DBP diastolic blood pressure; HDL high density lipoprotein; LDL low-density lipoprotein; SBP systolic blood pressure; Q quintile.

Supplemental Table 3. Baseline characteristics across quintiles of direct LDL-C among 346,686 UK Biobank participants.

Factor		Direct LDL-C mg/dL quintiles: mean (SD)					P-value
		Q1: 102.3 (11.9) n=69345	Q2: 126.0 (4.9) n=69415	Q3: 142.0 (4.50) n=69282	Q4: 158.9 (5.5) n=69307	Q5: 189.4 (17.6) n=69337	
Age	years	52.80 (8.39)	54.80 (8.19)	55.93 (7.93)	56.66 (7.68)	57.46 (7.37)	<0.001
Sex	Female	40797 (58.8%)	38816 (55.9%)	38072 (55.0%)	37992 (54.8%)	41364 (59.7%)	<0.001
	Male	28548 (41.2%)	30599 (44.1%)	31210 (45.0%)	31315 (45.2%)	27973 (40.3%)	
Ethnicity	White	64334 (92.8%)	65485 (94.3%)	65840 (95.0%)	66364 (95.8%)	66916 (96.5%)	<0.001
	Black	2008 (2.9%)	1227 (1.8%)	913 (1.3%)	792 (1.1%)	564 (0.8%)	
	South Asian	1136 (1.6%)	1109 (1.6%)	1029 (1.5%)	841 (1.2%)	668 (1.0%)	
	Other	1867 (2.7%)	1594 (2.3%)	1500 (2.2%)	1310 (1.9%)	1189 (1.7%)	
Smoking status	Never	62044 (89.5%)	62605 (90.2%)	62498 (90.2%)	62119 (89.6%)	61524 (88.7%)	<0.001
	Ever	7301 (10.5%)	6810 (9.8%)	6784 (9.8%)	7188 (10.4%)	7813 (11.3%)	
SBP	mmHg	133.89 (19.23)	137.27 (19.31)	139.29 (19.28)	141.03 (19.36)	143.02 (19.74)	<0.001
DBP	mmHg	79.78 (10.72)	81.46 (10.62)	82.49 (10.57)	83.43 (10.58)	84.38 (10.57)	<0.001
Antihypertensive medication		8405 (12.1%)	8710 (12.5%)	8858 (12.8%)	8753 (12.6%)	7850 (11.3%)	<0.001
Diabetes mellitus		2661 (3.8%)	1254 (1.8%)	891 (1.3%)	592 (0.9%)	511 (0.7%)	<0.001
Triglycerides	mg/dL	113.87 (71.05)	134.52 (78.51)	149.51 (83.07)	165.20 (86.26)	187.84 (91.16)	<0.001
Total cholesterol	mg/dL	177.09 (20.05)	206.08 (14.36)	225.50 (14.02)	246.15 (14.49)	284.39 (25.81)	<0.001

ApoB	g/L	0.77 (0.10)	0.94 (0.07)	1.05 (0.07)	1.17 (0.08)	1.39 (0.15)	<0.001
Friedewald LDL-C	mg/dL	97.80 (15.13)	122.84 (10.10)	139.65 (10.30)	157.45 (11.21)	190.27 (21.82)	<0.001
Martin/Hopkins LDL-C	mg/dL	99.26 (13.54)	124.78 (7.32)	141.69 (7.38)	159.59 (8.51)	191.86 (20.76)	<0.001
Non-HDL-C	mg/dL	120.06 (15.79)	149.09 (10.06)	168.78 (10.32)	189.63 (11.58)	226.90 (23.86)	<0.001
HDL-C	mg/dL	57.03 (15.25)	56.99 (14.83)	56.72 (14.39)	56.52 (13.79)	57.50 (12.96)	<0.001
ApoA1	g/L	1.54 (0.28)	1.55 (0.27)	1.55 (0.27)	1.55 (0.27)	1.56 (0.26)	<0.001

ApoA1 Apolipoprotein A1; ApoB Apolipoprotein B; CVD cardiovascular disease; DBP diastolic blood pressure; HDL high density lipoprotein; LDL low-density lipoprotein; SBP systolic blood pressure; Q quintile.

Supplemental Table 4. Baseline characteristics across quintiles of non-HDL-C among 346,686 UK Biobank participants.

Factor		Non-HDL-C cholesterol mg/dL quintiles: mean (SD)					P-value
		Q1: 119.0 (14.3) n=69350	Q2: 148.4 (6.2) n=69358	Q3: 168.5 (5.7) n=69344	Q4: 189.9 (7.0) n=69300	Q5: 228.6 (22.3) n=69334	
Age	years	52.71 (8.35)	54.90 (8.16)	56.04 (7.93)	56.75 (7.68)	57.25 (7.45)	<0.001
Sex	Female	42948 (61.9%)	39868 (57.5%)	38166 (55.0%)	37130 (53.6%)	38929 (56.1%)	<0.001
	Male	26402 (38.1%)	29490 (42.5%)	31178 (45.0%)	32170 (46.4%)	30405 (43.9%)	
Ethnicity	White	64354 (92.8%)	65462 (94.4%)	65869 (95.0%)	66328 (95.7%)	66926 (96.5%)	<0.001
	Black	2098 (3.0%)	1267 (1.8%)	892 (1.3%)	754 (1.1%)	493 (0.7%)	
	South Asian	1042 (1.5%)	1045 (1.5%)	1047 (1.5%)	921 (1.3%)	728 (1.0%)	
	Other	1856 (2.7%)	1584 (2.3%)	1536 (2.2%)	1297 (1.9%)	1187 (1.7%)	
Smoking status	Never	62416 (90.0%)	62736 (90.5%)	62558 (90.2%)	61975 (89.4%)	61105 (88.1%)	<0.001
	Ever	6934 (10.0%)	6622 (9.5%)	6786 (9.8%)	7325 (10.6%)	8229 (11.9%)	
SBP	mmHg	133.24 (19.15)	137.18 (19.32)	139.43 (19.30)	141.28 (19.27)	143.36 (19.58)	<0.001
DBP	mmHg	79.38 (10.64)	81.35 (10.59)	82.52 (10.56)	83.56 (10.51)	84.72 (10.56)	<0.001
Antihypertensive medication		7633 (11.0%)	8691 (12.5%)	8958 (12.9%)	9129 (13.2%)	8165 (11.8%)	<0.001
Diabetes mellitus		2427 (3.5%)	1252 (1.8%)	982 (1.4%)	682 (1.0%)	566 (0.8%)	<0.001
TG	mg/dL	99.97 (50.61)	124.38 (61.51)	145.36 (72.40)	169.45 (83.80)	211.78 (105.29)	<0.001
Total cholesterol	mg/dL	178.02 (20.96)	206.28 (15.87)	225.25 (15.36)	245.61 (15.29)	284.06 (26.04)	<0.001

ApoB	g/L	0.77 (0.10)	0.94 (0.07)	1.05 (0.07)	1.17 (0.08)	1.39 (0.15)	<0.001
Direct LDL-C	mg/dL	103.06 (12.69)	126.53 (7.45)	142.23 (7.77)	158.77 (9.11)	188.07 (19.24)	<0.001
Friedewald LDL-C	mg/dL	99.15 (15.75)	123.79 (12.43)	139.93 (13.63)	156.96 (15.15)	188.19 (24.23)	<0.001
Martin/Hopkins LDL-C	mg/dL	99.79 (13.89)	125.46 (8.71)	141.88 (9.70)	159.55 (11.23)	190.51 (22.56)	<0.001
HDL-C	mg/dL	58.99 (15.24)	57.85 (14.78)	56.74 (14.38)	55.68 (13.71)	55.49 (12.79)	<0.001
ApoA1	g/L	1.56 (0.28)	1.56 (0.28)	1.55 (0.27)	1.54 (0.26)	1.54 (0.26)	<0.001

ApoA1 Apolipoprotein A1; ApoB Apolipoprotein B; CVD cardiovascular disease; DBP diastolic blood pressure; HDL high density lipoprotein; LDL low-density lipoprotein; SBP systolic blood pressure; Q quintile.

Supplemental Table 5. Baseline characteristics across quintiles of HDL-C among 346,686 UK Biobank participants.

Factor		HDL-C mg/dL quintiles: mean (SD)					P-value
		Q1: 39.1 (4.2) n=69367	Q2: 48.2 (2.1) n=69423	Q3: 55.3 (2.1) n=69361	Q4: 63.5 (2.7) n=69261	Q5: 78.7 (8.6) n=69274	
Age	years	54.46 (8.35)	55.07 (8.24)	55.54 (8.08)	55.91 (7.95)	56.67 (7.60)	<0.001
Sex	Female	17959 (25.9%)	30379 (43.8%)	40703 (58.7%)	49865 (72.0%)	58135 (83.9%)	<0.001
	Male	51408 (74.1%)	39044 (56.2%)	28658 (41.3%)	19396 (28.0%)	11139 (16.1%)	
Ethnicity	White	64496 (93.0%)	65460 (94.3%)	65943 (95.1%)	66243 (95.6%)	66797 (96.4%)	<0.001
	Black	1137 (1.6%)	1249 (1.8%)	1084 (1.6%)	1105 (1.6%)	929 (1.3%)	
	South Asian	1837 (2.6%)	1126 (1.6%)	880 (1.3%)	575 (0.8%)	365 (0.5%)	
	Other	1897 (2.7%)	1588 (2.3%)	1454 (2.1%)	1338 (1.9%)	1183 (1.7%)	
Smoking status	Never	59259 (85.4%)	61476 (88.6%)	62619 (90.3%)	63226 (91.3%)	64210 (92.7%)	<0.001
	Ever	10108 (14.6%)	7947 (11.4%)	6742 (9.7%)	6035 (8.7%)	5064 (7.3%)	
SBP	mmHg	139.98 (18.48)	139.59 (19.26)	138.80 (19.67)	137.97 (20.10)	138.16 (20.54)	<0.001
DBP	mmHg	83.99 (10.56)	83.27 (10.68)	82.33 (10.72)	81.27 (10.72)	80.67 (10.62)	<0.001
Antihypertensive medication		11196 (16.1%)	9280 (13.4%)	8342 (12.0%)	7241 (10.5%)	6517 (9.4%)	<0.001
Diabetes mellitus		2561 (3.7%)	1337 (1.9%)	874 (1.3%)	639 (0.9%)	498 (0.7%)	<0.001
Triglycerides	mg/dL	213.90 (109.93)	168.74 (84.15)	144.08 (70.81)	122.95 (58.00)	101.11 (43.89)	<0.001
Total cholesterol	mg/dL	211.32 (38.30)	223.84 (39.72)	228.60 (40.34)	232.63 (39.59)	242.82 (38.65)	<0.001

ApoB	g/L	1.08 (0.22)	1.10 (0.24)	1.08 (0.24)	1.05 (0.23)	1.02 (0.22)	<0.001
Direct LDL-C	mg/dL	139.48 (28.90)	146.08 (31.03)	146.22 (32.12)	144.50 (32.03)	142.35 (31.41)	<0.001
Friedewald LDL-C	mg/dL	132.09 (32.28)	142.54 (33.53)	144.75 (34.62)	144.65 (34.93)	143.96 (35.11)	<0.001
Martin/Hopkins LDL-C	mg/dL	137.19 (30.83)	145.47 (33.05)	146.30 (34.43)	145.05 (34.76)	143.16 (34.84)	<0.001
Non-HDL-C	mg/dL	172.21 (37.51)	175.60 (39.67)	173.29 (40.34)	169.15 (39.58)	164.17 (38.21)	<0.001
ApoA1	g/L	1.23 (0.12)	1.40 (0.10)	1.53 (0.10)	1.67 (0.12)	1.93 (0.20)	<0.001

ApoA1 Apolipoprotein A1; ApoB Apolipoprotein B; CVD cardiovascular disease; DBP diastolic blood pressure; HDL high density lipoprotein; LDL low-density lipoprotein; SBP systolic blood pressure; Q quintile.

Supplemental Table 6. Baseline characteristics across quintiles of ApoA1 among 346,686 UK Biobank participants.

Factor		Apolipoprotein A1 g/L quintiles: mean (SD)					P-value
		Q1: 1.21 (0.09) n=69581	Q2: 1.39 (0.04) n=69382	Q3: 1.53 (0.04) n=69260	Q4: 1.67 (0.05) n=69382	Q5: 1.96 (0.17) n=69081	
Age	years	53.99 (8.36)	54.91 (8.24)	55.56 (8.05)	56.18 (7.87)	57.01 (7.55)	<0.001
Sex	Female	20328 (29.2%)	30732 (44.3%)	40060 (57.8%)	49045 (70.7%)	56876 (82.3%)	<0.001
	Male	49253 (70.8%)	38650 (55.7%)	29200 (42.2%)	20337 (29.3%)	12205 (17.7%)	
Ethnicity	White	64352 (92.5%)	65455 (94.3%)	65870 (95.1%)	66525 (95.9%)	66737 (96.6%)	<0.001
	Black	1373 (2.0%)	1265 (1.8%)	1104 (1.6%)	979 (1.4%)	783 (1.1%)	
	South Asian	1854 (2.7%)	1135 (1.6%)	848 (1.2%)	578 (0.8%)	368 (0.5%)	
	Other	2002 (2.9%)	1527 (2.2%)	1438 (2.1%)	1300 (1.9%)	1193 (1.7%)	
Smoking status	Never	59518 (85.5%)	61732 (89.0%)	62611 (90.4%)	63367 (91.3%)	63562 (92.0%)	<0.001
	Ever	10063 (14.5%)	7650 (11.0%)	6649 (9.6%)	6015 (8.7%)	5519 (8.0%)	
SBP	mmHg	138.34 (18.46)	138.87 (19.30)	138.78 (19.71)	138.74 (20.05)	139.77 (20.59)	<0.001
DBP	mmHg	83.11 (10.60)	82.82 (10.74)	82.34 (10.79)	81.72 (10.76)	81.54 (10.68)	<0.001
Antihypertensive medication		9865 (14.2%)	9043 (13.0%)	8379 (12.1%)	7776 (11.2%)	7513 (10.9%)	<0.001
Diabetes mellitus		2234 (3.2%)	1324 (1.9%)	1015 (1.5%)	750 (1.1%)	586 (0.8%)	<0.001
Triglycerides	mg/dL	190.80 (106.57)	163.39 (89.21)	146.37 (78.28)	131.87 (68.96)	118.22 (60.39)	<0.001
Total cholesterol	mg/dL	211.31 (40.19)	221.83 (39.53)	227.67 (39.12)	233.73 (38.31)	244.77 (38.24)	<0.001

ApoB	g/L	1.08 (0.23)	1.08 (0.24)	1.07 (0.23)	1.06 (0.23)	1.03 (0.22)	<0.001
Direct LDL-C	mg/dL	140.34 (30.87)	144.36 (31.28)	145.03 (31.46)	144.87 (31.21)	144.05 (31.06)	<0.001
Friedewald LDL-C	mg/dL	134.87 (33.94)	141.22 (33.99)	143.31 (34.24)	144.27 (34.32)	144.35 (34.83)	<0.001
Martin/Hopkins LDL-C	mg/dL	138.75 (32.83)	143.78 (33.43)	144.99 (33.87)	145.15 (33.97)	144.50 (34.35)	<0.001
Non-HDL-C	mg/dL	171.03 (39.43)	173.05 (39.75)	172.13 (39.66)	170.38 (38.96)	167.84 (38.34)	<0.001
HDL-C	mg/dL	40.28 (5.46)	48.79 (4.89)	55.54 (5.43)	63.35 (6.36)	76.93 (10.42)	<0.001

ApoA1 Apolipoprotein A1; ApoB Apolipoprotein B; CVD cardiovascular disease; DBP diastolic blood pressure; HDL high density lipoprotein; LDL low-density lipoprotein; SBP systolic blood pressure; Q quintile.

Supplemental Table 7. Distribution of baseline characteristics in UK Biobank participants according to whether or not they suffered a fatal CVD event (SCORE definition) during follow-up.

Factor		No fatal CVD (n=345,030)	Fatal CVD (n=1,656)	P-value
Age	years	55.50 (8.08)	60.89 (6.75)	<0.001
Sex	Female	196516 (57.0%)	525 (31.7%)	<0.001
	Male	148514 (43.0%)	1131 (68.3%)	
Ethnicity	White	327344 (94.9%)	1595 (96.3%)	0.035
	Black	5490 (1.6%)	14 (0.8%)	
	South Asian	4763 (1.4%)	20 (1.2%)	
	Other	7433 (2.2%)	27 (1.6%)	
Smoking status	Never	309514 (89.7%)	1276 (77.1%)	<0.001
	Ever	35516 (10.3%)	380 (22.9%)	
SBP	mmHg	138.85 (19.62)	148.41 (21.90)	<0.001
DBP	mmHg	82.29 (10.72)	85.41 (12.28)	<0.001
Antihypertensive medication		42111 (12.2%)	465 (28.1%)	<0.001
Diabetes		5803 (1.7%)	106 (6.4%)	<0.001
Triglycerides	mg/dL	150.09 (86.05)	169.09 (93.97)	<0.001
Total cholesterol	mg/dL	227.83 (40.66)	228.38 (42.70)	0.58
ApoB,	g/L	1.07 (0.23)	1.11 (0.24)	<0.001
Direct LDL-C	mg/dL	143.71 (31.22)	146.79 (32.43)	<0.001
Friedewald LDL-C	mg/dL	141.59 (34.44)	143.32 (36.60)	<0.001
Martin/Hopkins LDL-C	mg/dL	143.42 (33.77)	146.22 (35.44)	0.041
Non-HDL-C	mg/dL	170.86 (39.26)	176.16 (40.88)	<0.001
HDL-C	mg/dL	56.97 (14.27)	52.22 (14.07)	
ApoA1	gl/L	1.55 (0.27)	1.47 (0.27)	<0.001

ApoA1 Apolipoprotein A1; ApoB Apolipoprotein B; CVD cardiovascular disease; DBP diastolic blood pressure; HDL high density lipoprotein; LDL low-density lipoprotein; SBP systolic blood pressure; Q quintile.

Supplemental Table 8. Adjusted (age, sex, ethnicity, SBP, DBP, antihypertensive medication, diabetes, smoking, and baseline CVD) associations of different lipid and lipoprotein measures with composite CVD, by quintile, amongst those participants who reported taking statins (n=68,649).

	Quintiles					
	Q1	Q2	Q3	Q4	Q5	P-trend
ApoB g/L, Mean (SD)	0.62 (0.07)	0.75 (0.03)	0.84 (0.03)	0.95 (0.03)	1.16 (0.15)	
Range	0.40-0.70	0.71-0.80	0.80-0.89	0.89-1.01	1.01-2.00	
Events/total	744/13767	713/13697	691/13757	657/13762	710/13666	
Rate per 1000 person years (95% CI)	6.41 (5.97-6.89)	6.16 (5.72-6.63)	5.93 (5.50-6.38)	5.60 (5.19-6.04)	6.13 (5.69-6.59)	
Adjusted HR (95% CI)	0.87 (0.79-0.97)	0.96 (0.87-1.07)	1 (REF)	1.03 (0.92-1.14)	1.28 (1.15-1.42)	<0.001
Direct LDL-C, mg/dL Mean (SD)	75.13 (8.66)	92.95 (3.74)	105.20 (3.46)	118.66 (4.57)	147.52 (20.23)	
Range	10.67-86.20	86.23-99.23	99.27-111.25	111.29-127.22	127.26-274.44	
Events/total	796/13768	746/13715	655/13722	666/13745	652/13699	
Rate per 1000 person years (95% CI)	6.90 (6.44-7.40)	6.44 (5.99-6.91)	5.63 (5.21-6.07)	5.68 (5.26-6.13)	5.59 (5.18-6.04)	
Adjusted HR (95% CI)	0.91 (0.82-1.01)	1.01 (0.91-1.11)	1 (REF)	1.13 (1.01-1.26)	1.29 (1.16-1.44)	<0.001
Friedewald LDL-C, mg/dL Mean (SD)	59.95 (10.83)	80 (4.18)	94.39(3.88)	109.34 (5.07)	141.42 (22.79)	
Range	0.88-73.09	73.10-87.73	87.73-101.18	101.18-118.85	118.85-286.58	
Events/total	843/13730	700/13730	688/13730	624/13730	660/13729	
Rate per 1000 person years (95% CI)	7.33 (6.85-7.84)	6.02 (5.59-6.48)	5.91 (5.48-6.37)	5.33 (4.93-5.77)	5.65 (5.24-6.10)	
Adjusted HR (95% CI)	0.92 (0.83-1.02)	0.91 (0.82-1.02)	1 (REF)	1.04 (0.93-1.16)	1.27 (1.14-1.42)	<0.001
Martin/Hopkins LDL-C, mg/dL	68.17 (9.32)	87.04 (4.05)	100.15 (3.63)	114.47 (4.93)	145.72 (22.17)	

Mean (SD)						
Range	5.96-79.79	79.79-93.82	83.82-106.51	106.51-123.85	123.86-286.68	
Events/total	793/13730	763/13730	672/13730	629/13730	658/13729	
Rate per 1000 person years (95% CI)	6.89 (6.43-7.39)	6.57 (6.12-7.05)	5.78 (5.35-6.23)	5.37 (4.96-5.81)	5.63 (5.22-6.08)	
Adjusted HR (95% CI)	0.89 (0.80-0.98)	1.01 (0.91-1.12)	1 (REF)	1.05 (0.94-1.17)	1.29 (1.16-1.44)	<0.001
Non-HDL, mg/dL Mean (SD)	89.70 (10.46)	111.67 (4.70)	127.04 (4.41)	144.14 (5.82)	180.84 (25.60)	
Range	46.83-103.13	103.17-119.45	119.45-134.77	134.80-155.07	155.07-380.24	
Events/total	789/13732	703/13743	662/13732	666/13723	695/13719	
Rate per 1000 person years (95% CI)	6.85 (6.39-7.35)	6.05 (5.62-6.51)	5.68 (5.26-6.13)	5.69 (5.27-6.14)	5.96 (5.53-6.42)	
Adjusted HR (95% CI)	0.93 (0.84-1.03)	0.96 (0.86-1.06)	1 (REF)	1.11 (0.99-1.23)	1.32 (1.19-1.47)	<0.001
HDL cholesterol, mg/dL Mean (SD)	34.57 (3.57)	42.36 (1.75)	48.33 (1.80)	55.58 (2.51)	70.78 (9.38)	
Range	9.74-39.17	39.21-45.28	45.32-51.55	51.59-60.32	60.36-129.66	
Events/total	1060/13789	789/13736	657/13681	529/13746	480/13697	
Rate per 1000 person years (95% CI)	9.22 (8.69-9.80)	6.78 (6.33-7.27)	5.64 (5.23-6.09)	4.51 (4.14-4.91)	4.11 (3.76-4.50)	
Adjusted HR (95% CI)	1.20 (1.08-1.32)	1.06 (0.96-1.18)	1 (REF)	0.89 (0.79-0.99)	0.93 (0.83-1.05)	<0.001
ApoA1, g/L Mean (SD)	1.15 (0.09)	1.33 (0.04)	1.45 (0.04)	1.60 (0.05)	1.88 (0.18)	
Range	0.42-1.26	1.26-1.39	1.39-1.52	1.52-1.68	1.69-2.50	
Events/total	1079/13743	820/13847	638/13635	533/13721	445/13703	
Rate per 1000 person years (95% CI)	9.43 (8.88-10.01)	7.02 (6.56-7.52)	5.49 (5.08-5.93)	4.55 (4.18-4.95)	3.80 (3.47-4.17)	
Adjusted HR (95% CI)	1.29 (1.16-1.42)	1.15 (1.04-1.28)	1 (REF)	0.94 (0.83-1.05)	0.89 (0.79-1.01)	<0.001

ApoA1 Apolipoprotein A1; ApoB Apolipoprotein B; CI confidence interval; DBP diastolic blood pressure; HDL high density lipoprotein; LDL low-density lipoprotein; SBP systolic blood pressure; Q quintile.

Supplemental Table 9. Adjusted (age, sex, ethnicity, SBP, DBP, antihypertensive medication, diabetes, smoking, and baseline CVD) associations of different lipid and lipoprotein measures with composite CVD using a linear model, amongst those participants who reported taking statins with or without baseline CVD (n=68,649).

	**Adjusted** HR per 1SD increase	P-value
ApoB	1.16 (1.12-1.20)	<0.001
Direct LDL-C,	1.15 (1.11-1.19)	<0.001
Friedewald LDL-C	1.14 (1.10-1.18)	<0.001
Martin/Hopkins LDL-C	1.15 (1.11-1.19)	<0.001
Non-HDL-C	1.16 (1.12-1.20)	<0.001
HDL-C	0.89 (0.86-0.93)	<0.001
ApoA1	0.87 (0.83-0.90)	<0.001

ApoA1 Apolipoprotein A1; ApoB Apolipoprotein B; HDL high density lipoprotein; LDL low-density lipoprotein; SD standard deviation.

Supplemental Table 10. Adjusted (age, sex, ethnicity, SBP, DBP, antihypertensive medication, diabetes, and smoking) associations of different lipid and lipoprotein measures with composite CVD, by quintile of the distribution among the discordant population who were not taking statins and had no history of CVD at baseline (i.e. >=10% absolute difference in baseline percentile of direct LDL and ApoB, n=63,520).

	Quintiles					
	Q1	Q2	Q3	Q4	Q5	P- trend
ApoB g/L, Mean (SD)	0.87 (0.07)	0.99 (0.02)	1.06 (0.02)	1.13 (0.02)	1.26 (0.08)	
Range	0.46-0.95	0.95-1.02	1.03-1.10	1.10-1.18	1.18-1.80	
Events/total	197/12769	210/12671	249/12702	223/12691	364/12687	
Rate per 1000 person years (95% CI)	1.76 (1.53-2.02)	1.90 (1.66-2.17)	2.24 (1.98-2.54)	2.01 (1.76-2.29)	3.31 (2.99-3.67)	
Adjusted HR (95% CI)	0.88 (0.73-1.06)	0.89 (0.74-1.07)	1 (REF)	0.88 (0.73-1.05)	1.29 (1.10-1.52)	<0.001
Direct LDL-C, mg/dL Mean (SD)	117.46 (8.19)	132.80 (3.04)	142.59 (1.73)	152.54 (3.14)	168.95 (9.25)	
Range	64.08-127.30	127.34-137.86	137.90-147.33	147.37-158.24	158.28-261.18	
Events/total	266/12706	253/12704	227/12702	266/12717	231/12691	
Rate per 1000 person years (95% CI)	2.41 (2.14-2.72)	2.28 (2.02-2.58)	2.04 (1.79-2.33)	2.39 (2.12-2.69)	2.07 (1.82-2.36)	
Adjusted HR (95% CI)	1.05 (0.88-1.26)	1.10 (0.92-1.31)	1 (REF)	1.16 (0.98-1.39)	1.09 (0.90-1.30)	0.538
Friedewald LDL-C, mg/dL Mean (SD)	107.92 (11.16)	127.56 (3.73)	139.09 (3.16)	150.73 (3.69)	170.48 (11.30)	
Range	42.58-120.67	120.67-133.65	133.65-144.60	144.60-157.55	157.55-286.24	
Events/total	295/12704	250/12704	241/12704	238/12704	219/12704	
Rate per 1000 person years (95% CI)	2.68 (2.39-3.00)	2.26 (1.99-2.55)	2.17 (1.91-2.46)	2.14 (1.89-2.43)	1.96 (1.72-2.24)	
Adjusted HR (95% CI)	1.01 (0.85-1.20)	1.01 (0.84-1.20)	1 (REF)	1.01 (0.85-1.21)	1.01 (0.84-1.22)	0.985
Martin/Hopkins LDL-C, mg/dL	113.93 (9.08)	130.84 (3.32)	141.47 (2.99)	152.41 (3.44)	170.82 (10.92)	

Mean (SD)						
Range	46.69-124.71	124.71-136.36	136.36-146.66	146.67-158.70	158.71-320.22	
Events/total	276/12704	253/12704	230/12704	252/12704	232/12704	
Rate per 1000 person years (95% CI)	2.50 (2.22-2.82)	2.28 (2.02-2.58)	2.07 (1.82-2.36)	2.27 (2.00-2.57)	2.08 (1.83-2.37)	
Adjusted HR (95% CI)	1.05 (0.88-1.25)	1.07 (0.90-1.28)	1 (REF)	1.10 (0.92-1.31	1.09 (0.91-1.31)	0.584
Non-HDL, mg/dL Mean (SD)	139.15 (9.54)	157.76 (3.81)	170.12 (3.49)	183.02 (4.17)	205.18 (13.56)	
Range	74.32-150.81	150.81-164.12	164.15-176.18	176.18-190.72	190.72-409.79	
Events/total	249/12707	218/12709	234/12708	277/12694	265/12702	
Rate per 1000 person years (95% CI)	2.26 (1.99-2.55)	1.96 (1.72-2.24)	2.11 (1.85-2.39)	2.50 (2.22-2.81)	2.38 (2.11-2.69)	
Adjusted HR (95% CI)	1.04 (0.87-1.25)	0.96 (0.79-1.15)	1 (REF)	1.13 (0.95-1.35)	1.10 (0.92-1.31)	0.163
HDL cholesterol, mg/dL Mean (SD)	35.43 (3.76)	44.72 (2.45)	53.85 (2.80)	64.55 (3.43)	82.40 (9.47)	
Range	13.42-40.56	40.60-49.11	49.15-58.82	58.86-70.84	70.88-141.42	
Events/total	419/12760	319/12649	206/12736	168/12682	131/12693	
Rate per 1000 person years (95% CI)	3.80 (3.45-4.18)	2.90 (2.60-3.24)	1.85 (1.61-2.12)	1.51 (1.29-1.75)	1.18 (0.99-1.40)	
Adjusted HR (95% CI)	1.67 (1.41-1.99)	1.36 (1.14-1.63)	1 (REF)	0.93 (0.75-1.14)	0.77 (0.61-0.96)	<0.001
ApoA1, g/L Mean (SD)	1.14 (0.09)	1.35 (0.05)	1.52 (0.05)	1.70 (0.06)	2.03 (0.17)	
Range	0.42-1.26	1.26-1.43	1.43-1.60	1.61-1.81	1.81-2.50	
Events/total	421/12753	306/12714	196/12666	172/12724	148/12663	
Rate per 1000 person years (95% CI)	3.82 (3.47-4.20)	2.77 (2.47-3.10)	1.77 (1.54-2.03)	1.53 (1.32-1.78)	1.33 (1.14-1.57)	
Adjusted HR (95% CI)	1.83 (1.54-2.18)	1.39 (1.16-1.67)	1 (REF)	0.99 (0.80-1.21)	0.89 (0.71-1.10)	<0.001

ApoA1 Apolipoprotein A1; ApoB Apolipoprotein B; CI confidence interval; CVD cardiovascular disease; DBP diastolic blood pressure; HDL high density lipoprotein; LDL low-density lipoprotein; SBP systolic blood pressure; SD standard deviation; Q quintile.

Supplemental Table 11. Adjusted (age, sex, ethnicity, SBP, DBP, antihypertensive medication, diabetes, and smoking) associations of different lipid and lipoprotein measures with composite CVD using a linear model among the discordant population who were not taking statins and had no history of CVD at baseline (i.e. >=10% absolute difference in baseline percentile of direct LDL and ApoB, n=63520).

	**Adjusted** HR per 1SD increase	P-value
ApoB	1.23 (1.12-1.35)	<0.001
Direct LDL-C,	1.00 (0.91-1.10)	0.97
Friedewald LDL-C	1.00 (0.91-1.09)	0.94
Martin/Hopkins LDL-C	1.00 (0.91-1.10)	0.95
Non-HDL-C	1.08 (0.98-1.18)	0.11
HDL -C	0.78 (0.74-0.83)	<0.001
ApoA1	0.78 (0.73-0.83)	<0.001

ApoA1 Apolipoprotein A1; ApoB Apolipoprotein B; HDL high density lipoprotein; HR hazard ratio; LDL low-density lipoprotein; SD standard deviation.

Supplemental Table 12. Stratified and adjusted (age, sex, ethnicity, SBP, DBP, antihypertensive medication, diabetes, and smoking) associations of different lipid and lipoprotein measures with composite CVD using a linear model (n=346,686).

Subgroup	N events/N participants	HR per 1SD increase, (95% CI)						
		ApoB	Direct LDL-c	Friedewald LDL-c	Martin/Hopkins LDL-c	Non-HDL-c	HDL-c	ApoA1
Women	2145/197041	1.14 (1.10-1.19)	1.11 (1.07-1.16)	1.08 (1.04-1.13)	1.10 (1.06-1.15)	1.13 (1.09-1.18)	0.82 (0.79-0.86)	0.84 (0.80-0.88)
Men	4071/149645	1.26 (1.22-1.30)	1.23 (1.19-1.27)	1.21 (1.17-1.25)	1.22 (1.19-1.26)	1.24 (1.20-1.28)	0.84 (0.81-0.87)	0.82 (0.79-0.84)
		p-interaction <0.0001	p-interaction <0.0001	p-interaction <0.0001	p-interaction <0.0001	p-interaction <0.0001	p-interaction 0.78	p-interaction 0.030
Under 50	661/94021	1.32 (1.23-1.42)	1.29 (1.19-1.38)	1.24 (1.15-1.34)	1.27 (1.18-1.36)	1.31 (1.22-1.41)	0.79 (0.72-0.87)	0.81 (0.74-0.89)
50-59	1810/121383	1.31 (1.26-1.37)	1.28 (1.23-1.34)	1.25 (1.20-1.31)	1.28 (1.22-1.34)	1.30 (1.24-1.36)	0.78 (0.74-0.83)	0.79 (0.75-0.84)
60+	3745/131282	1.14 (1.11-1.18)	1.12 (1.08-1.16)	1.10 (1.07-1.14)	1.12 (1.08-1.16)	1.13 (1.10-1.17)	0.83 (0.80-0.86)	0.81 (0.78-0.84)
		p-interaction <0.0001	p-interaction <0.0001	p-interaction <0.0001	p-interaction <0.0001	p-interaction <0.0001	p-interaction 0.0002	p-interaction 0.018
Ever smoker	1214/35896	1.29 (1.22-1.36)	1.26 (1.19-1.33)	1.24 (1.17-1.31)	1.26 (1.19-1.33)	1.26 (1.19-1.33)	0.83 (0.77-0.88)	0.81 (0.76-0.86)
Never smoker	5002/310790	1.21 (1.18-1.24)	1.18 (1.15-1.22)	1.15 (1.12-1.19)	1.17 (1.14-1.21)	1.20 (1.17-1.24)	0.81 (0.78-0.84)	0.81 (0.78-0.83)
		p-interaction 0.057	p-interaction 0.024	p-interaction 0.006	p-interaction 0.0009	p-interaction 0.113	p-interaction 0.152	p-interaction 0.331
Diabetes patients*	246/5909	1.17 (1.03-1.33)	1.17 (1.03-1.33)	1.18 (1.03-1.35)	1.17 (1.03-1.33)	1.17 (1.03-1.33)	0.85 (0.74-0.99)	0.82 (0.71-0.95)
No diabetes	5970/340777	1.23 (1.20-1.26)	1.20 (1.17-1.23)	1.17 (1.14-1.20)	1.19 (1.16-1.22)	1.22 (1.18-1.25)	0.81 (0.79-0.84)	0.81 (0.78-0.83)
		p-interaction 0.61	p-interaction 0.21	p-interaction 0.49	p-interaction 0.87	p-interaction 0.57	p-interaction 0.28	p-interaction 0.17
High Triglycerides**	1978/76581	1.25 (1.20-1.31)	1.23 (1.17-1.29)	1.22 (1.16-1.28)	1.23 (1.18-1.29)	1.24 (1.18-1.29)	0.90 (0.86-0.95)	0.86 (0.82-0.91)
Low Triglycerides**	4238/270105	1.17 (1.14-1.21)	1.14 (1.11-1.18)	1.14 (1.10-1.17)	1.14 (1.11-1.18)	1.15 (1.12-1.19)	0.82 (0.79-0.85)	0.81 (0.78-0.84)

		p-interaction <0.0001	p-interaction <0.0001	p-interaction <0.0001	p-interaction <0.0001	p-interaction <0.0001	p-interaction 0.69	p-interaction 0.41

*Self-reported diabetes or those who reported using insulin, **Cut-off 195 mg/dL (2.2 mmol/L)

ApoA1 Apolipoprotein A1; ApoB Apolipoprotein B; CI confidence interval; CVD cardiovascular disease; DBP diastolic blood pressure; HDL high density lipoprotein; HR hazard ratio; LDL low-density lipoprotein; SBP systolic blood pressure; SD standard deviation; Q quintile.

Supplemental Table 13. Improvement in goodness of model fit for prediction of incident fatal and non-fatal CVD under different combinations of lipids and lipoproteins added to classical risk factors.

Model lipids/ proteins	Altered by	AIC	BIC	ΔAIC	ΔBIC	%Δ AIC	%Δ BIC
Classical risk factors* with no lipids		153192	153299.5				
	+total cholesterol & HDL-C	152809	152938.1	-383.00	-361.40	-0.25	-0.24
	+ApoA1 & ApoB	152769.9	152898.9	-422.10	-400.60	-0.28	-0.26
Classical risk factors* with total cholesterol & HDL-C		152809	152938.1				
	+ ApoA1 & ApoB	152771	152921.6	-38.00	-16.50	-0.02	-0.01
	+direct LDL-C	152804	152943.8	-5.00	5.70	0.00	0.00
	+Friedewald LDL-C	152801.1	152940.9	-7.90	2.80	-0.01	0.00
	+Martin/Hopkins LDL-C	152800.2	152940.1	-8.80	2.00	-0.01	0.00
	+ApoB alone	152788.6	152928.4	-20.40	-9.70	-0.01	-0.01

*Classical risk factors: age, sex, ethnicity, SBP, DBP, antihypertensive medication, diabetes, and smoking.

AIC Akaike Information Criterion; ApoA1 Apolipoprotein A1; ApoB Apolipoprotein B; BIC Bayesian Information Criterion; HDL high density lipoprotein; LDL low-density lipoprotein;.

Supplemental Table 14. Categorical Net Reclassification Index (NRI) across the 7.5% 10 year risk boundary by comparison of different lipid/lipoprotein panels in the risk score, in the overall cohort without baseline CVD and not taking statins (n=346,686)

Comparator	Addition	Overall NRI (95% CI)	Case NRI (95%CI)	Non-case NRI (95% CI)
**Classical risk factors* with no lipids**				
	+Total cholesterol & HDL-C	+0.60% (-0.03, +1.29%)	+0.79% (+0.16, +1.48%)	-0.18% (-0.22, -0.14%)
	+ApoA1 & ApoB	+0.60% (-0.05, +1.25%)	+0.79% (+0.14, +1.44%)	-0.18% (-0.22, -0.15%)
**Classical risk factors* with total cholesterol & HDL-C**				
	+ ApoB	+0.14% (-0.17, +0.50%)	+0.16% (-0.16, +0.50%)	-0.01% (-0.03, 0.00%)
	+direct LDL-C	+0.13% (-0.11, +0.41%)	+0.13% (-0.10, +0.40%)	0.00% (-0.01, +0.01%)
	+Freidewald LDL-C	+0.13% (-0.12, +0.42%)	+0.13% (-0.12, +0.41%)	0.0% (-0.01, +0.02%)
	+Martin/Hopkins LDL-C	+0.13% (-0.13, +0.42%)	+0.13% (-0.13, +0.43%)	0.00% (-0.01, +0.02%)

*Classical risk factors: age, sex, ethnicity, SBP, DBP, antihypertensive medication, diabetes, and smoking.

ApoA1 Apolipoprotein A1; ApoB Apolipoprotein B; CI confidence interval; HDL high density lipoprotein; LDL low-density lipoprotein.

Supplemental Figure 1. Kaplan-Meier plots of event-free survival rate of composite fatal/nonfatal CVD by quartiles of total cholesterol (a), Apolipoprotein B (b), direct low density lipoprotein-C (c), Non-high-density lipoprotein-C (d), Friedewald LDL-C (e), Martin/Hopkins LDL-C (f), HDL-C (g) and Apolipoprotein A1 (h). Quartile 1 is low, quartile 5 is high.

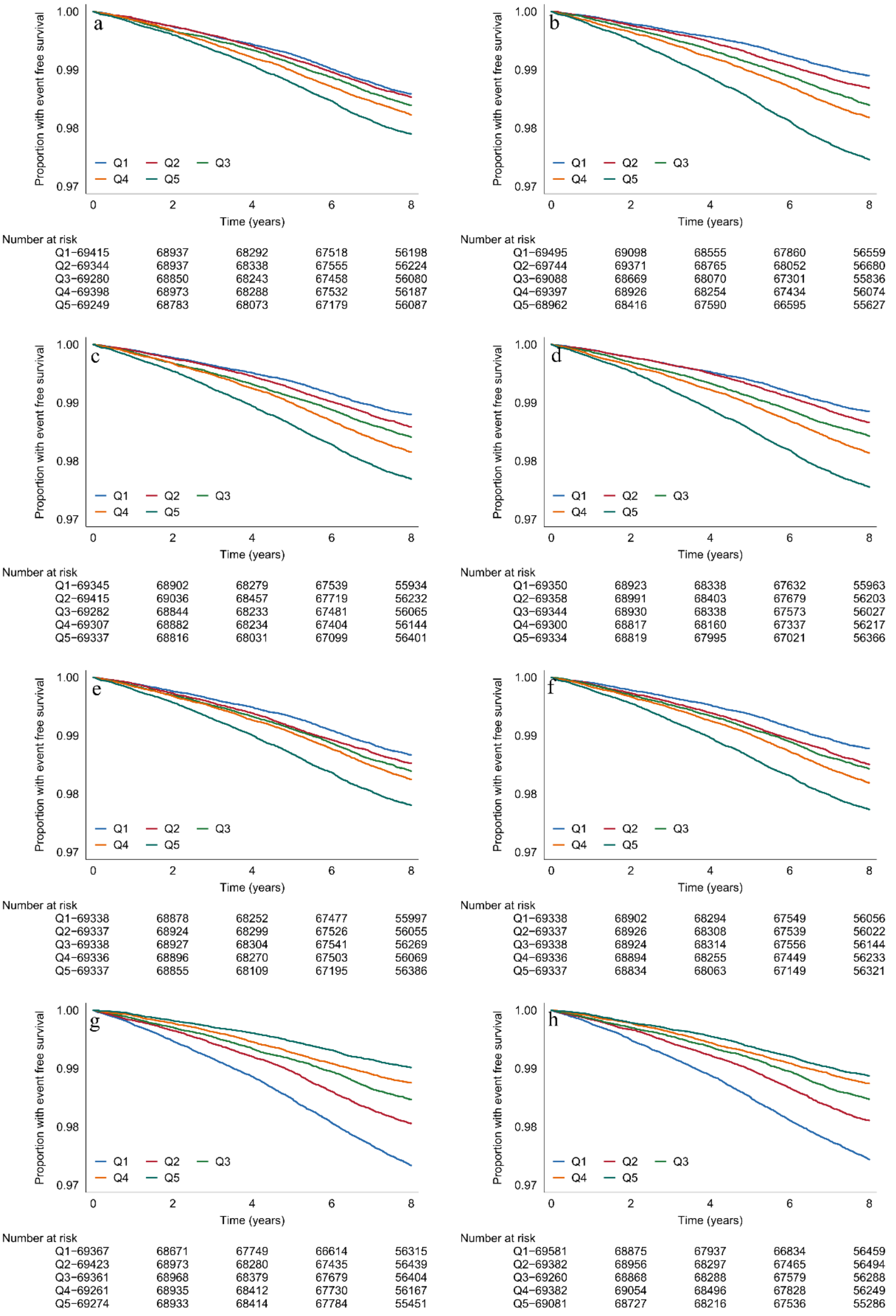

Supplemental Figure 2. Kaplan-Meier plot of event-free survival rate of fatal CVD by quartiles of total cholesterol (a), ApoB (b), direct LDL-C (c), Non-HDL-C (d), Friedewald LDL-C (e), Martin/Hopkins LDL-C (f), HDL-C (g) and ApoA1 (h). Quartile 1 is low, quartile 5 is high.

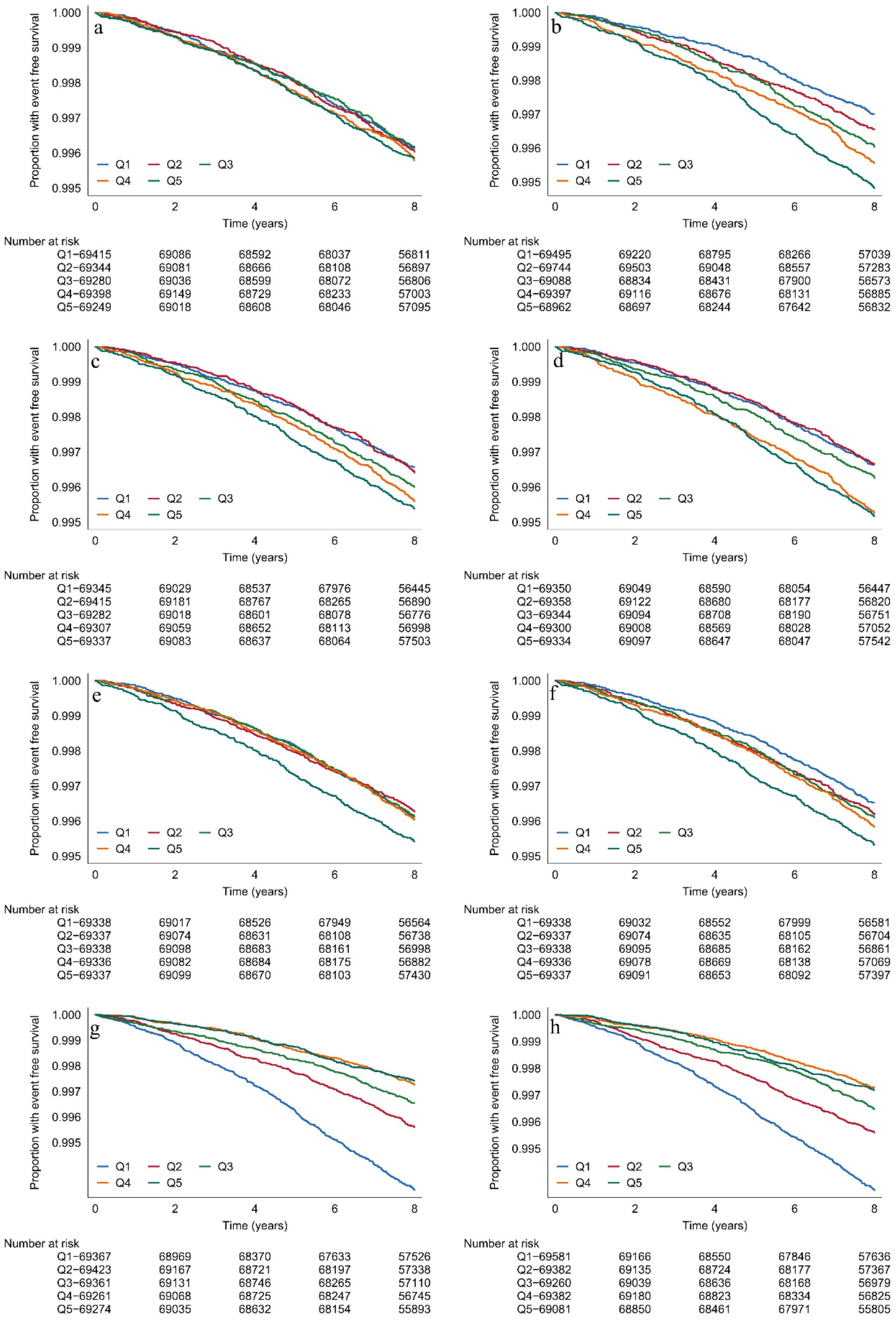

Supplemental Figure 3. Adjusted (for age, sex, SBP, smoking) association of total cholesterol, ApoB, direct LDL-C, non-HDL-C, calculated LDL-C, HDL-C, and ApoA1 with fatal cardiovascular disease events (n=1,656 events).

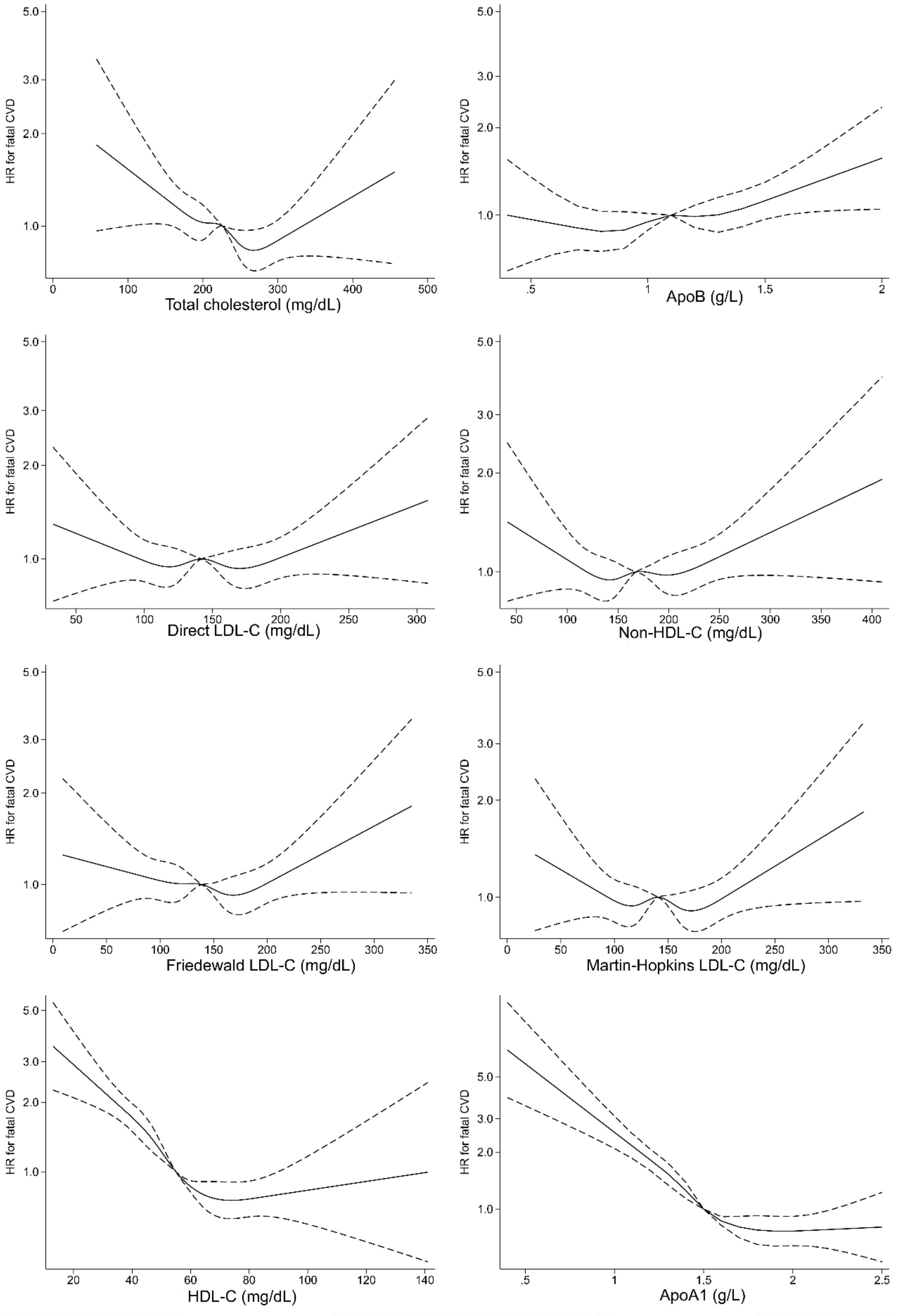

Supplemental Figure 4. Adjusted (age, sex, SBP and smoking) association of total cholesterol, ApoB, direct LDL-C, non-HDL-C, calculated LDL-C, HDL-C, and ApoA1 with fatal CVD events after excluding first 2 years of follow-up (n=1,440 events).

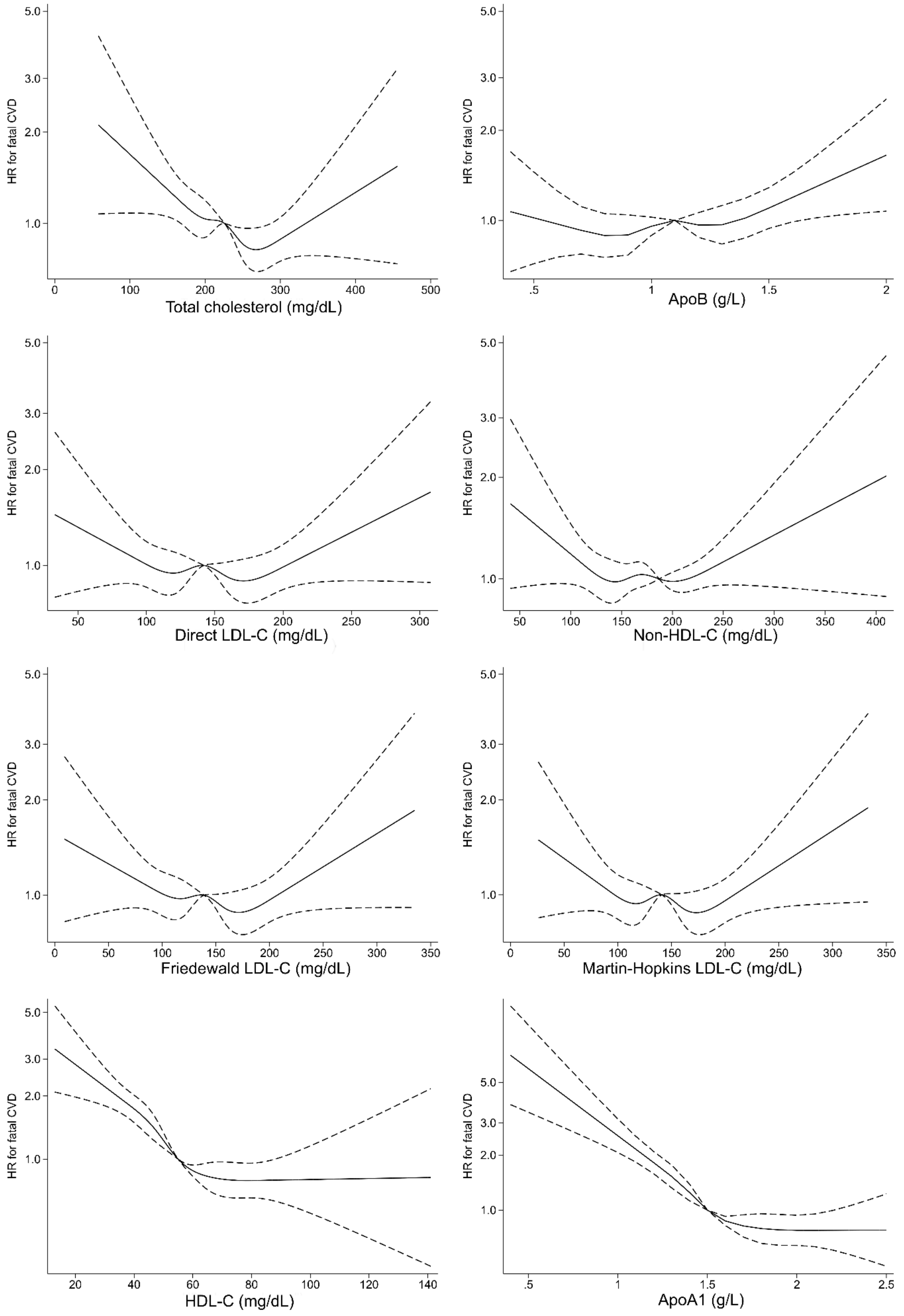

Supplemental Figure 5. Change in C-Index for prediction of incident fatal CVD upon addition or substitution of lipids and apolipoproteins to classical CVD risk factors in those with no baseline CVD and not taking a statin.

	N participants	n cases	C-index (95%CI)	Change in C-index (95%CI)
**Either/or model**	346,686	1,656		
Classical risk factors			0.7813 (0.7708, 0.7919)	
Classical risk factors plus total cholesterol & HDL-C				+0.0036 (0.0015, 0.0056)
Classical risk factors plus ApoA1 & ApoB				+0.0045 (0.0021, 0.0069)
**Additive model**	346,686	1,656		
Classical risk factors plus total cholesterol & HDL-C			0.7849 (0.7745, 0.7954)	
plus ApoA1 & ApoB				+0.0011 (-0.0003, 0.0024)
plus direct LDL-C				-0.0001 (-0.0007, 0.0005)
plus Friedewald LDL-C				+0.0001 (-0.0006, 0.0008)
plus Martin/Hopkins LDL-C				+0.0001 (-0.0008, 0.0011)
plus ApoB only				+0.0002 (-0.0006, 0.0011)

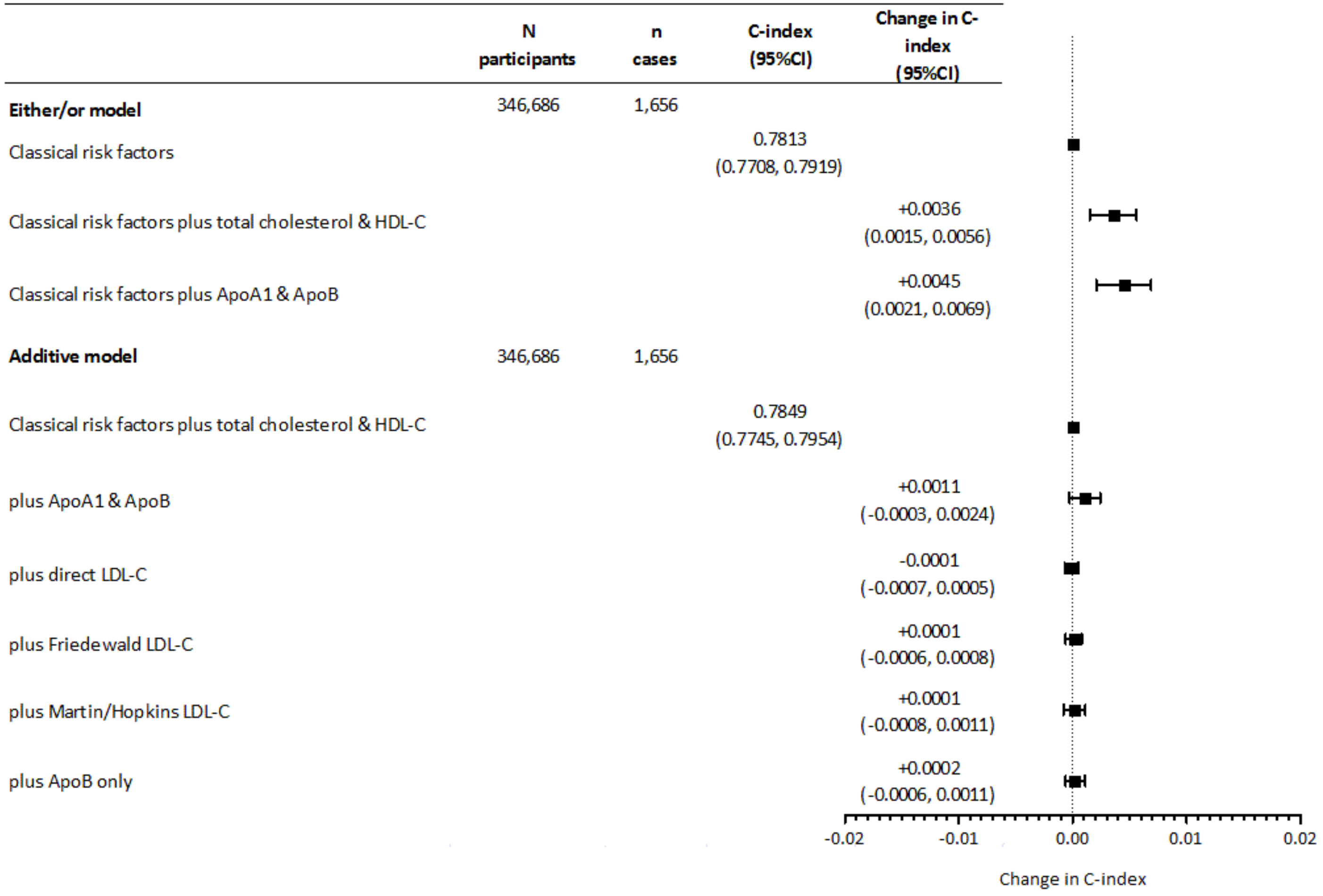

Supplemental Figure 6. Change in C-Index for prediction of incident fatal/non-fatal cardiovascular disease (CVD) on addition of different lipid and lipoprotein combinations among those taking a statin (classical risk factors: age, sex, ethnicity, systolic blood pressure, diastolic blood pressure, antihypertensive medication, diabetes, smoking, and baseline CVD)

	N participants	n cases	C-index (95%CI)	Change in C-index (95%CI)
**Either/or model**	68,649	3,514		
Classical risk factors			0.7118 (0.7033, 0.7203)	
Classical risk factors plus non-HDL				+0.0030 (0.0012, 0.0048)
Classical risk factors plus direct LDL-C				+0.0026 (0.0010, 0.0043)
Classical risk factors plus Friedewald LDL-C				+0.0024 (0.0008, 0.0040)
Classical risk factors plus Martin/Hopkins LDL-C				+0.0026 (0.0010, 0.0043)
Classical risk factors plus ApoB				+0.0030 (0.0011, 0.0048)
**Additive model**	68,649	3,514		
Classical risk factors plus non-HDL			0.7148 (0.7063, 0.7233)	
Classical risk factors plus non-HDL & direct LDl-C				+0.0001 (-0.0002, 0.0003)
Classical risk factors plus non-HDL & Friedewald LDL-C				+0.0000 (0.0000, 0.0000)
Classical risk factors plus non-HDL & Martin/Hopkins LDL-C				+0.0000 (-0.0001, 0.0001)
Classical risk factors plus non-HDL & ApoB				+0.0001 (-0.0003, 0.0004)

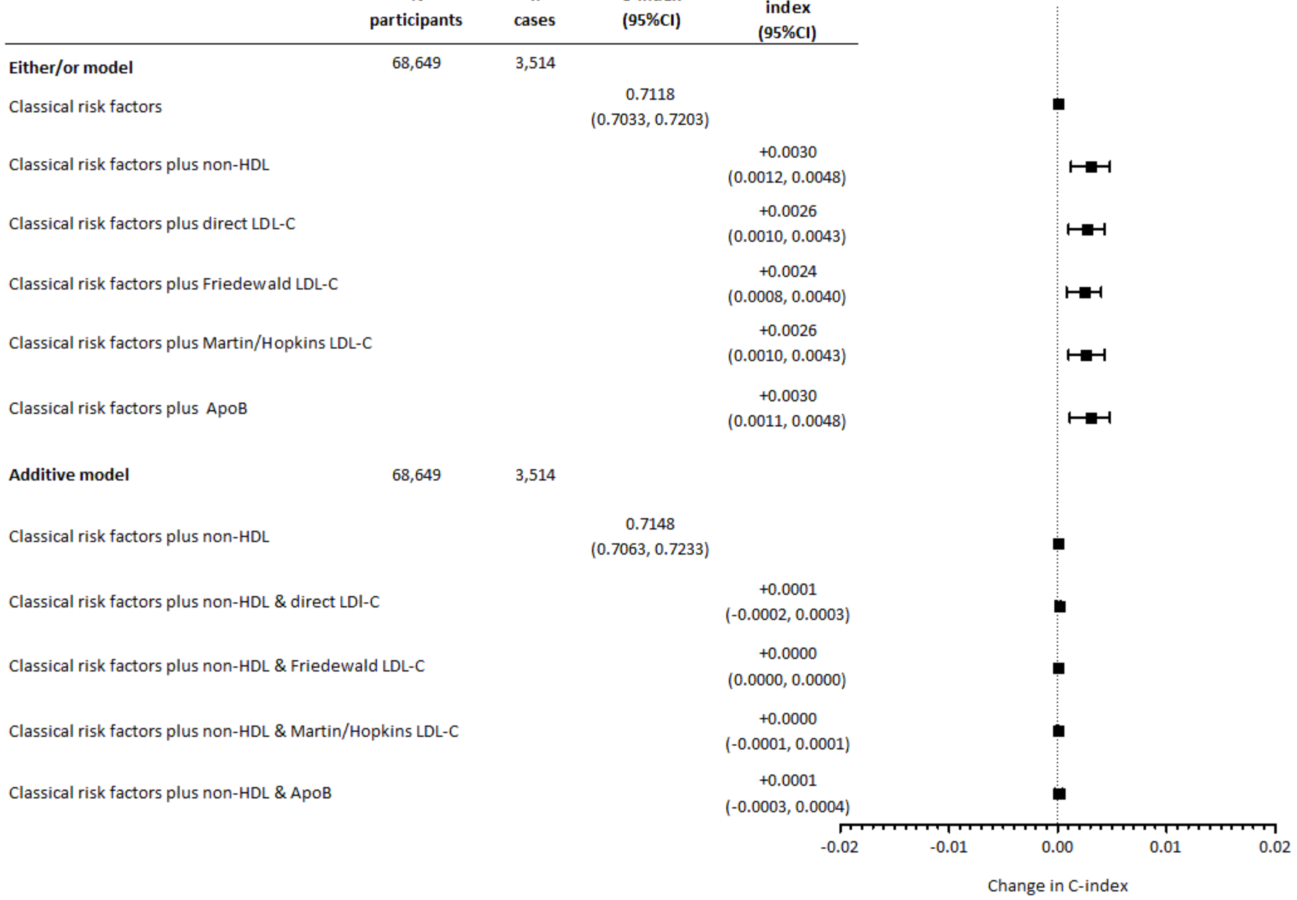

Supplement references

1. UK Biobank showcase. Blood Sample Collection, Processing and Transport. 04/15/2011. https://biobank.ctsu.ox.ac.uk/showcase/docs/Bloodsample.pdf (accessed 04/17/19)
2. Elliott P, Peakman TC, UK Biobank. The UK Biobank sample handling and storage protocol for the collection, processing and archiving of human blood and urine. *Int J Epidemiol.* 2008;37:234–244.
3. UK Biobank showcase. Biospeciments manual: Collection of biological samples, processing and storage. 04/08/2011 https://biobank.ctsu.ox.ac.uk/crystal/docs/BioSampleProc.pdf (accessed 04/17/19)
4. UK Biobank showcase. Companion Document to Accompany Serum Biomarker Data. Version 1.0. 03/11/2019 https://biobank.ctsu.ox.ac.uk/showcase/docs/serum_biochemistry.pdf (accessed 04/17/19)
5. UK Biobank showcase. Biomarker assay quality procedures: approaches used to minimise systematic and random errors. Version 2.0. 04/02/2019 https://biobank.ctsu.ox.ac.uk/showcase/docs/biomarker_issues.pdf (accessed 04/17/19)
6. Martin SS, Giugliano RP, Murphy SA, Wasserman SM, Stein EA, Ceška R, López-Miranda J, Georgiev B, Lorenzatti AJ, Tikkanen MJ, Sever PS, Keech AC, Pedersen TR, Sabatine MS. Comparison of Low-Density Lipoprotein Cholesterol Assessment by Martin/Hopkins Estimation, Friedewald Estimation, and Preparative Ultracentrifugation: Insights From the FOURIER Trial. *JAMA Cardiol*. 2018;3:749-753.
